# Scanning single molecule localization microscopy (scanSMLM) for super-resolution volume imaging

**DOI:** 10.1038/s42003-023-05364-2

**Published:** 2023-10-17

**Authors:** Jigmi Basumatary, Neptune Baro, Prakash Joshi, Partha Pratim Mondal

**Affiliations:** 1 Nanobioimaging Laboratory, Department of Instrumentation and Applied Physics, Indian Institute of Science, Bangalore, 560012 India; 2 Centre for Cryogenic Technology, Indian Institute of Science, Bangalore, 560012 India

**Keywords:** Wide-field fluorescence microscopy, Fluorescence imaging, Super-resolution microscopy

## Abstract

Over the last decade, single-molecule localization microscopy (SMLM) has developed into a set of powerful techniques that have improved spatial resolution over diffraction-limited microscopy and demonstrated the ability to resolve biological features down to a few tens of nanometers. We introduce a single molecule-based scanning SMLM (*scanSMLM*) system that enables rapid volume imaging. Along with epi-illumination, the system employs a scanning-based 4f detection for volume imaging. The 4f system comprises a combination of an electrically-tunable lens and high NA detection objective lens. By rapidly changing the aperture (or equivalently the focus) of an electrically-tunable lens (ETL) in a 4f detection system, the selectivity of the axial object plane is achieved, for which the image forms in the image/detector plane. So, in principle, one can scan the object volume by just altering the aperture of ETL. Two schemes were adopted to carry out volume imaging: cyclic scan and conventional scan. The cyclic scheme scans the volume in each scan cycle, whereas plane-wise scanning is performed in the conventional scheme. Hence, the cyclic scan ensures uniform dwell time on each frame during data collection, thereby evenly distributing photobleaching throughout the cell volume. With a minimal change in the system hardware (requiring the addition of an ETL lens and related electronics for step-voltage generation) in the existing SMLM system, volume scanning (along the z-axis) can be achieved. To calibrate and derive critical system parameters, we imaged fluorescent beads embedded in a gel-matrix 3D block as a test sample. Subsequently, *scanSMLM* is employed to visualize the architecture of actin-filaments and the distribution of Meos-Tom20 molecules on the mitochondrial membrane. The technique is further exploited to understand the clustering of Hemagglutinin (HA) protein single molecules in a transfected cell for studying Influenza-A disease progression. The system, for the first time, enabled 3D visualization of HA distribution that revealed HA cluster formation spanning the entire cell volume, post 24 hrs of transfection. Critical biophysical parameters related to HA clusters (density, the number of HA molecules per cluster, axial span, fraction of clustered molecules, and others) are also determined, giving an unprecedented insight into Influenza-A disease progression at the single-molecule level.

## Abstract (web summary)

A combination of an electrically-tunable lens and an objective lens in a 4f detection system allows for scanning single molecule localization microscopy for super-resolution volume imaging.

## Introduction

To be able to visualize single molecules in a cell volume is an incredible feat. This gives a plethora of information related to local physiology and helps in deciphering the underlying biological mechanism. A true super-resolution volume imaging system has numerous benefits ranging from organelle-level investigation to whole cell volume analysis, benefiting studies related to collective dynamics (e.g. clustering) during infection and unraveling fundamental biology occurring at single molecule level. This is seldom possible with the existing SMLM techniques. So, the development of single molecule-based volume imaging techniques is of great interest.

In the last decade, SMLM techniques and its variants have taken a big leap with resolution approaching sub-10 nm regime and temporal resolution of few milliseconds. Recent advances in SMLM such as, Probabilistic Optically-Selective Single-molecule Imaging Based Localization Encoded (POSSIBLE), Minimal Photon Fluxes (MINFLUX), direct Stochastic Optical Reconstruction Microscopy (dSTORM), Structured Illumination based Point Localization Estimator (SIMPLE) and Repetitive Optical Selective Exposure (ROSE) have shown sub-10 nm resolution^1–7^. With the advent of molecules that have high quantum yield and large molar extinction coefficient (e.g., pamCherry, Dendra2, Cy5, and Alexa Fluor 647), the resolution has bettered by an order. None-the-less the fact that super-resolution is restricted to single plane of interest has somewhat handicapped the progress of SMLM. However, few techniques have shown multi-plane imaging capability which is a step closer to volume imaging. In this regard, the integration of light sheet and super-resolution have shown great promise^8^. Specifically, 3D super-resolution microscopy is reported by pairing tilted light sheet with long-range PSF^9^. The technique allows determination of the location of individual molecules detected throughout the volume excited by the light sheet. Moreover, the technique allows 3D single particle tracking (SPT) and single molecule SR imaging in 3D^10,11^. Among techniques that are truly capable of 3D imaging are, biplane fluorescence PhotoActivation Localization Microscopy (biplane-fPALM) and double-helix PSF^12–14^. Off-late, localization microscopy over large volumes (upto 20 μm) has become important which is addressed by integrating lattice light sheet with cell-permeable chemical probes^9^. It may be noted that electrically tunable lenses are used in quantitative phase microscopy that facilitate dynamic TIE phase imaging^15^. Another promising technique is 3D Stochastic Optical Reconstruction Microscopy (STORM) that uses optical astigmatism to determine both lateral and axial positions of individual molecules^16^. Specifically, multicolor STORM imaging of large volume has enabled ultrathin sectioning of ganglion cells to understand nanoscale co-organization of AMPA receptors and its spatial correlation with neuroligin-1^17–19^. Techniques primarily based on REversible Saturable OpticaL Fluorescence Transitions (RESOLFT) microscopy have shown 3D imaging of single molecules organization in a cell^20^. The technique has reported volumetric imaging of exosomes labeled with CD63-rsEGFP2 in live U2OS cells. Other techniques predominately based on evanescent light such as SMILE have demonstrated volume imaging capabilities in a single cell^21^. Although these techniques are promising, but none are free of constrains, often complex, difficult to adapt, use toxic bio-incompatible probes and are primarily designed for 2D imaging.

Although super-resolution techniques have advanced in recent years, they lack extensive volume imaging capability, suffer from strong background, and are inherently slow. So, techniques that enable fast imaging, large field-of-view and 3D super-resolution are particularly interesting. Among others, the existing 3D imaging techniques can be primarily categorized as (1) methods that reconstruct 3D volume by stacking 2D images and (2) techniques that are inherently 3D and achieve super-resolution in all three dimensions. For example, three-dimensional STORM can generate 3D super-resolved images by localizing both the axial and lateral positions of single molecules. Another technique that can perform 3D super-resolution imaging of thick specimens at large penetration depths (several micrometers) is bifocal plane imaging (BP-fPALM)^12,16^. Two recent techniques that can address these issues are 3D STORM and DNA-PAINT^22,23^. To extend beyond standard 2D super-resolution imaging, 3D STORM uses objective-based focal plane scanning and astigmatism to achieve whole cell imaging. The technique enabled 3D imaging of mitochondrial networks in mammalian cells. Another distinct technique is 3D fluorogenic DNA points accumulation for imaging in nanoscale topography (DNA-PAINT), that uses astigmatic detection for realizing 3D imaging^23^. This has facilitated fast astigmatic 3D fluorogenic DNA-PAINT imaging. The technique has the added advantage of performing simultaneous two-color imaging. Another pressing issue is achieving super-resolution imaging at large penetration depths primarily due to deteriorating PSF shape and aberrations caused by refractive index mismatch and scattering regions in a sample. In another development, Vytautas Navikas et al. have employed remote focussing that requires wavefront correction in an adaptive optics approach^24^. The technique has the distinct ability to correct aberrations and enables the engineering of PSF shape for better localization of emitters at large depths.

Classical SMLM techniques involve extracting single molecule signatures from a large set of recorded frames collected at video-rate. Subsequently, the bright spots are conveniently modeled by a 2D Gaussian, from which the centroid and number of photons are determined. These parameters serve as the basis to determine its position and size (governed by localization precision, ${\Delta }_{lp}^{2}=s/\sqrt{N}+{q}^{2}/12N+8\pi {s}^{4}{b}^{2}/{q}^{2}{N}^{2}$), where, *s* is the diffraction-limited PSF, *N* as the number of detected photons, *q* is the pixel size, and *b* is the noise respectively^25^). Most of the state-of-the-art single molecule super-resolution technique such as fPALM/PALM, STORM^26–33^ use these parameters to super-resolve features. Over the years, many important variants have emerged that have harnessed its potential in a wide range of research disciplines, and have enabled studies that were earlier thought to be nearly impossible. Specifically, the last decade has seen many variants including, super-resolution optical fluctuation imaging (SOFI)^34^, points accumulation for imaging in nanoscale topography (PAINT)^35,36^, simultaneous multiplane imaging-based localization encoded (SMILE)^21,37^, individual molecule localization-selective plane illumination microscopy (IML-SPIM)^8^, MINFLUX^7^, POSSIBLE microscopy^1^ and others^38–45^. Another class of super-resolution techniques is based on REversible Saturable OpticaL Fluorescence Transitions (RESOLFT) concept, such as, stimulated emission depletion (STED) and ground-state depletion microscopy (GSDIM) that have shown impressive resolution beyond diffraction limit^46,47^. But seldom, super-resolution techniques based on single molecule techniques have demonstrated the ability to map single molecules in a cell volume. Therefore, methods that facilitate visualization of single molecules in a cell volume are highly desirable. Such techniques are expected to expand the reach of super-resolution technique beyond the traditional super-resolution imaging.

In this article, we propose a scanning single-molecule localization microscopy (scanSMLM) that enabled axial scanning for realizing a super-resolution volume imaging. The technique belongs to methods that reconstruct 3D volume by stacking super-resolved 2D images. The technique employs a dedicated 4f detection system that comprise of electrically focal-length tunable lens (ETL) which facilitate focusing of fluorescence originating from the object planes on to the fixed image plane (detector plane). This is possible due to the ability of electrically tunable lens to alter its aperture, thereby facilitating both divergent and convergent rays (originating from off-focal z-planes of the specimen) to focus on the camera (image/detector plane). First, the technique is demonstrated on fluorescent bead sample (beads embedded in a Agarose gel-matrix). Subsequently, it is used to visualize the architecture of actin-filaments, and the organization of single molecules on mitochondrial network in a cell volume. This is followed by imaging single molecule (HA) clusters that are known to form during Influenza A viral infection. In this respect, a patent for working scanSMLM system along with associated optical design details is filed in India patent office^48^.

## Results

A single molecule super-resolution microscopy system is developed for volume imaging. The technique uses a dedicated 4f detection sub-system where an electrically tunable lens (ETL) is employed for image formation. A rapid change in the focal length of the ETL allows scanning in the object plane, thereby enabling imaging of multiple specimen planes. In addition, magnification optics are employed in the detection beam path to attain a magnification of approximately 280*X*. The images of single molecule PSFs in 10 chosen planes are recorded using two different scanning schemes (cyclic and conventional). Images are then processed to identify single molecules, which are stacked together to reconstruct the entire cell volume. The details are discussed in the methods section.

We study the Influenza type-A model that involves dynamic clustering of the glycoprotein Hemagglutinin (HA) in transfected NIH3T3 cells. HA protein is a glycoprotein found on the surface of influenza viruses, which is responsible for virus adhesion and entry into the host cell. Post viral entry in the host cytoplasm, the HA protein aggregates into HA clusters resulting in the onset of viral infection (Influenza A)^49–51^. It may be noted that HA clustering is a key process directly correlated with viral infectivity rate. Thus, understanding the HA molecular interactions at the single molecule level is essential to understand the mechanism of the HA accumulation process for designing an appropriate strategy to prevent HA clustering. For the HA clustering mechanism study by scanSMLM system, the NIH3T3 cells (seeding density, 104 cells/mL) were transfected with Dendra2HA plasmid, cultured for 24 h, and fixed with 2% PFA (as detailed in the “Methods” section) for superresolution imaging.

To better understand single molecule dynamics, it is imperative to know the photophysics of photoactivable probe. The photoactivable Dendra2 molecule involves the conversion between two states (on/off) mediated by triplet state. Accordingly, the priming or excitation of the anionic cis-chromophore populates the S1-state^52^. The de-population occurs via either fluorescence pathway or low-yield intersystem crossing to the lowest triplet state, T1. It may be noted that the mechanism involving conversion of primed state strongly relies on the creation of a triplet intermediate state. This results in fluorescence intermittencies such as triplet state transitions (popularly known as blinking) that are observed in many molecules. In general, several photoswitchable/photoactivable fluorescent dyes/proteins show blinking timescale of a few tens of milliseconds (e.g., Alexa555 has a timescale of 22 ± 6*ms* in deoxygenated PBS buffer^53^, and Dendra2 has an average blinking rate (illumination at 561 nm with an excitation power of 14 mW in PBS buffer of pH 7.4 at lab temperature (25 degrees)) of 23.1 ± 1.9*s*^−1^^54^). This necessitates that the images be acquired between 35−40 frames/sec. While a significant fraction of photoswitches is non-fluorescent, a small subset is stochastically activated and localized. The molecules can be localized to high precision by fitting a Gaussian function ($G(\mu ,\sigma ) \sim {e}^{-{(x-\mu )}^{2}/2{\sigma }^{2}}$, with *μ* and *σ* being the mean and standard deviation, respectively) to their point spread function and determining the number of collected photons. This leads to high localization precision (${\Delta }_{lp}^{2}=s/\sqrt{N}+{q}^{2}/12N+8\pi {s}^{4}{b}^{2}/{q}^{2}{N}^{2}$) for single molecules that are essential to render a super-resolution map of the specimen^25^. In our case, pixel size is *q* = 59 nm, and the average noise is *b* = 3.992 photons (see Supplementary note 11 and supplementary fig. 22). SMLM techniques requires the collection of several hundred frames containing single molecule signatures to reconstruct a single super-resolved 2D image. Here, we propose a 3D imaging technique (scanSMLM) to map single molecule in the entire cell volume. Specifically, we employed a cyclic scan that involves evenly scanning all the specimen layers/planes in a periodic manner. This scanning configuration brings uniformity across the planes and avoids long dwell time on a single plane as done in the conventional SMLM.

### The scanSMLM system

**Fig. 1:**
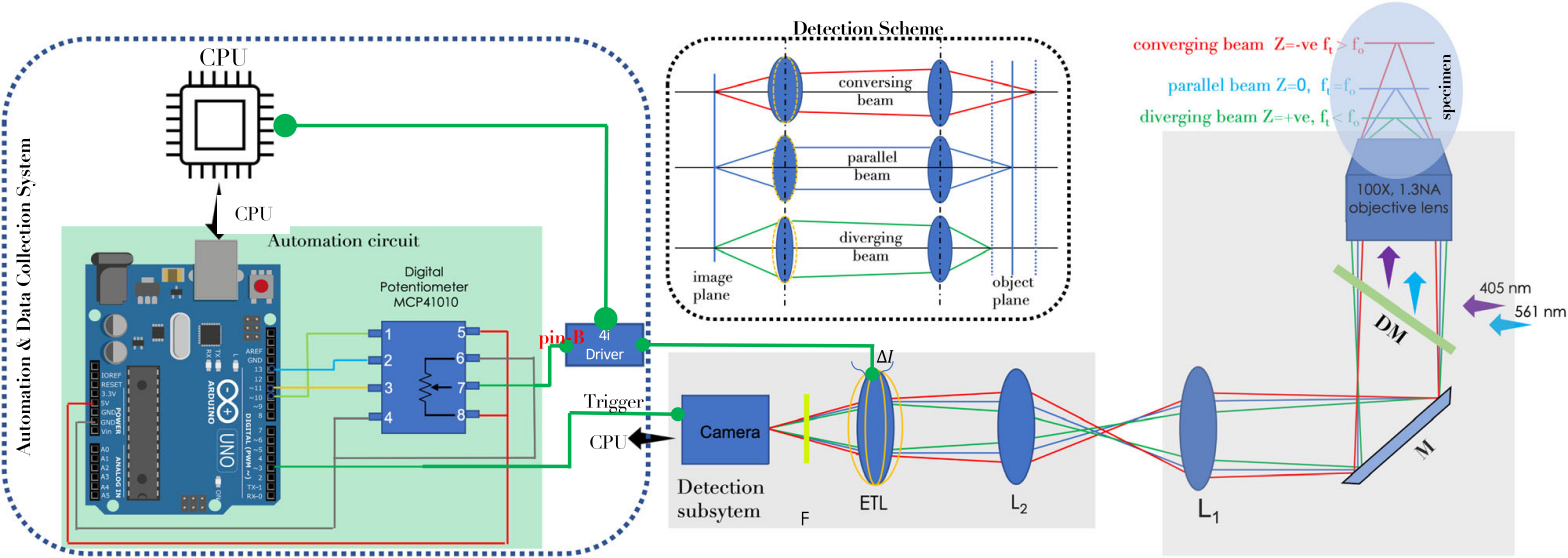
Schematic diagram of the proposed scanSMLM super-resolution microscopy. The illumination sub-system consists of two light sources: activation laser (405 nm) and excitation laser (561 nm). The beams are combined by a dichroic mirror (DM) and focused on the specimen by a high NA objective lens (100X, 1.3 NA). The fluorescence from the single molecules (in the specimen) are collected by the objective and directed to the EMCCD detector. On its way, the light is filtered by a set of filters (*F*). Additional magnification is introduced by a combination of biconvex and electrically tunable lens. The electrical lens driver 4*i* is used to control the focal power of ETLs. Discrete voltage step generated using digital potentiometer is applied on pin B of 4i hardware driver which controls the lens current set into firmware. The process of voltage generation, ETL tuning and camera are synchronized via Ardino (https://www.electronicscomp.com/mcp41010-10k-digital-potentiometer-with-spi-interface-ic). Additional details can be found in Supplementary note 1.

The schematic diagram of the proposed scanSMLM system is shown in Fig. 1. The illumination is achieved by a couple of lasers (405 nm light for activation and 561 nm light for excitation) and a high NA objective (Olympus, 100X objective, 1.3 NA). The emission from the focal plane (shown by blue rays) is collected by the objective lens and reflected by dichroic mirror to the a series of lenses that magnifies the image. Electrically tunable lens (ETL) has a fixed focal length at a specific set current value that corresponds to image formation of *z* = 0 plane. Now by increasing/decreasing current the aperture of ETL changes which focus on conversing/diverging rays originating from off-focal planes to the detector/image plane. Aperture change is effected by changing current through the ETL which is controlled by an external voltage. So, the image formed at the detector plane is that of off-focal planes of the object. Hence, by just changing the external voltage, one can achieve z-scanning in the object plane. This is schematically shown in the inset of Fig. 1 (see supplementary figs. 1 and 2). Rapid scanning of z-planes in the object plane is achieved by a 4*i* driver and an automation circuit. The electrical lens driver 4i helps the user to control the focal of ETL precisely. The lens current set into the firmware can be controlled by an analog applied voltage (0–5V) on the hardware Pin -B (see, Fig. 1). The applied voltage is linearly mapped in firmware to the range defined by the lower and upper current limits in the hardware configuration tab of Lens Driver Controller. The discrete step voltages are generated using digital potentiometer (MCP41010) via Arduino (microcontroller; see, Fig. 1). The actual optical system along with electronics and related automation unit are discussed in Supplementary note 1 (see supplementary figs. 3–5).

In the present study, the recording is carried out using a sensitive EMCCD camera (Andor 897iXon Ultra). The data is obtained at an exposure time of 30 ms with an EM gain of 250. The images are collected at full-frame (512 × 512). The laser’s power is optimized, with the activation (405 nm) and excitation (561 nm) laser intensity of 3 μW and 14 mW, respectively.

### The scanning scheme

**Fig. 2:**
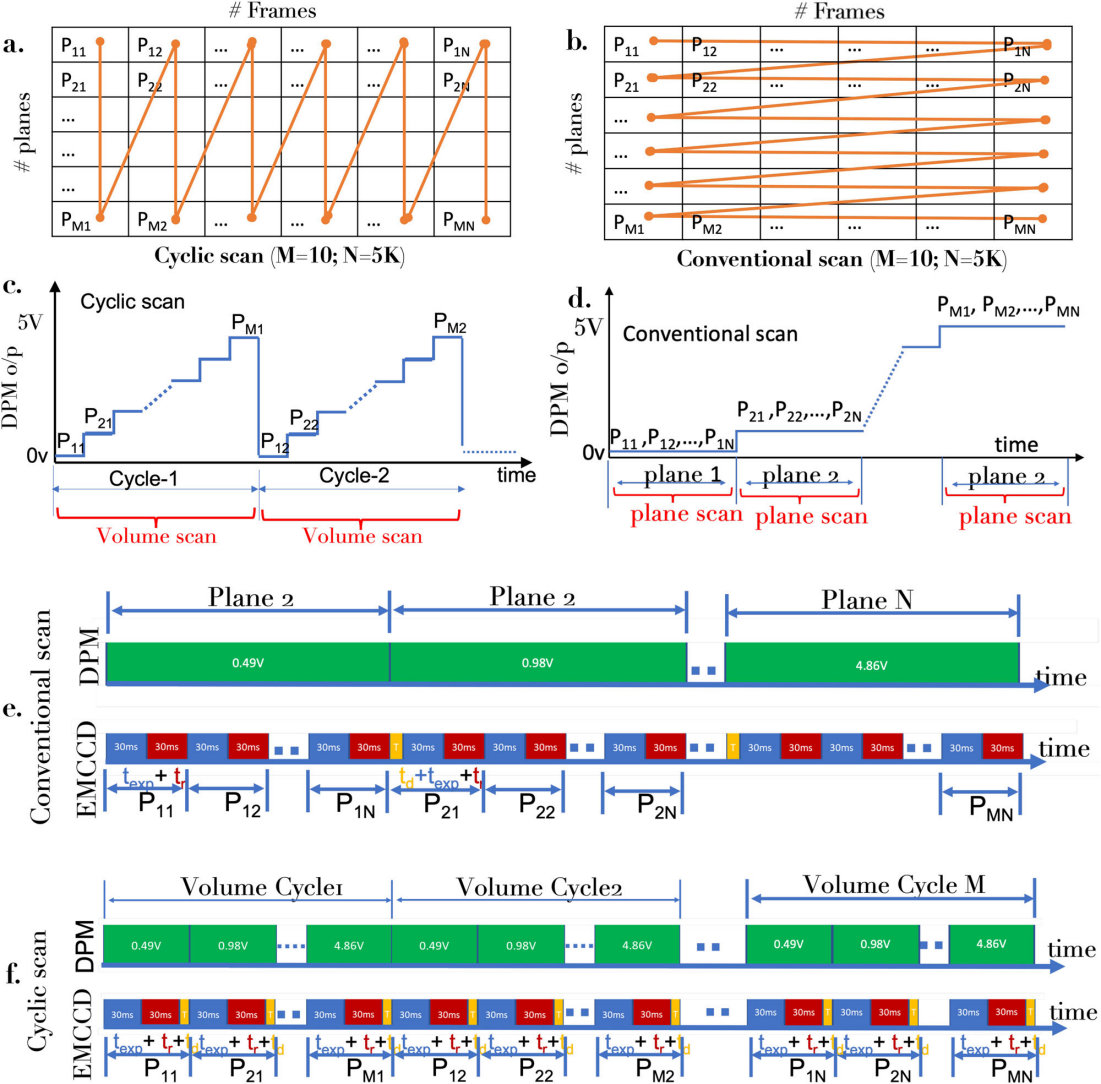
Volume scanning schemes. **a** Cyclic Scanning, (**b**) Conventional scanning. The element *p*_*ij*_ represents image of *i*^*th*^ plane during *j*^*th*^ cycle. **c** Generated step voltage for cyclic scan volume-wise, while (**d**) shows conventional scan plane-wise. In cyclic scanning mode, one frame of diffraction-limited single-molecule image is recorded for one step voltage, whereas in conventional scanning mode, all the single molecules frames are collected at a single step-voltage for a particular plane before proceeding to the next plane. **e**, **f** The sequencing of exposure time, *t*_exp_ ~ 5 ms; readout time, *t*_r_, and ETL response delay, *t*_d_ for volume and plane scanning cycles employed in cyclic and conventional scanning, respectively. The time synchronization of EMCCD camera and DPM discreet output step voltage generation is also displayed. Additional details can be found in Supplementary note 2.

To facilitate bias-free scanning of all the object planes/layers, a cyclic scan technique is adopted as shown in Fig. 2a. The table displays *#*frame versus *#*planes with each element *P*_*ij*_ corresponding to the *i*^*th*^ plane undergoing *j*^*th*^ scan. In the cyclic scan scheme, all the planes are treated equally with all of them undergoing first round of interrogation (activation and excitation). This is represented by *P*_*i*1_ element in the scan-matrix. Similar process is carried out to acquire other scan elements column-wise i.e., *P*_*i*1_ followed by *P*_*i*2_ and so on till *P*_*iN*_, where *N* represents the total number of realization of a single plane. On the other hand, traditional SMLM (see, Fig. 2b) mostly follow conventional row-wise scanning of planes with first plane illuminated *N* number of times. This is represented by *P*_1*j*_. Sequentially, data for other planes are collected i.e, first plane elements *P*_1*j*_ followed by second plane elements *P*_2*j*_ till the last plane *P*_*Mj*_ is collected. So, the existing SMLM techniques continuously exposes a single specimen plane for prolonged time, whereas scanSMLM equipped with cyclic scanning evenly exposes all the planes. In addition, existing SMLM scanning biases against the planes and hence cannot account for uniformity. The corresponding digital potentiometer (DPM) output (that linearly maps the current range set in firmware) versus time diagram are also shown for scanSMLM and conventional SMLM in Fig. 2c, d. In addition, synchronized time line of EMCCD detector (exposure and readout time), DPM o/p and ETL tuning delays are also shown for volume acquisition cycles in Fig. 2e. For comparison, conventional scan employed in traditional SMLM for collecting data plane-wise along with DPM voltage (for selecting specific plane) is also displayed (see Fig. 2f). More details related to scanning schemes and imaging protocol can be found in Supplementary note 2 and supplementary figs. 6 and 7.

### Optimization of scanSMLM System

**Fig. 3:**
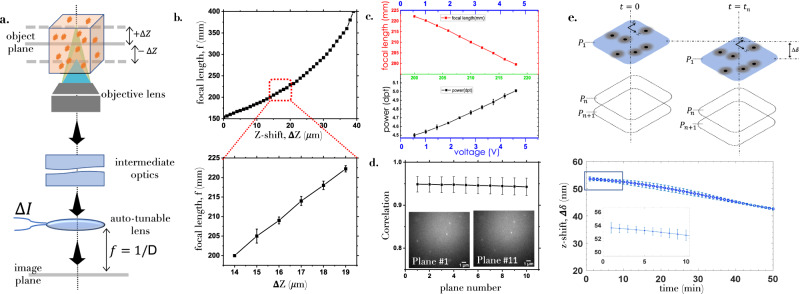
Characterization of scanSMLM system. **a** Schematic of detection sub-system reveals the connection between shift in object plane (Δ*Z*) and image formation (at *f*) by the electrically-tunable lens in the detector plane (image plane). **b** The characteristics of z-shift (Δ*Z*) versus focal length (*f*) along with its operating range of 0−4.5 μm (beginning from 14.0 μm) in the specimen (see, enlarged section). **c** The corresponding plot relating power/focal length (with an offset of 150 mm), and current/voltage. **d** The correlation between *P*_*i*,*j*_ and *P*_*i*+10,*j*_ image of fluorescent beads of size ~170 nm (embedded in agarose gel-matrix) in a cyclic scan. The correlation between them is found to be >94% demonstrating high repeatability of the planes. See Supplementary video 1 and 2, that shows the accuracy of cyclic scanning method. **e** Drift-correction along the axial *z*-axis indicates a stage-drift (Δ*δ*) of 0.2 nm/min. Additional details can be found in Supplementary note 3.

For accurate data acquisition, the system needs to be calibrated across the illumination volume and repeatability need to be ascertained. Based on the *Z*-resolution (≈500 nm) of the inverted microscope, we have chosen to work with 10 planes. The calibration is carried out with the mechanical knob of the microscope. Subsequently, the specimen planes are calibrated with the focal length of the electrically tunable lens as shown in Fig. 3a, b along with the enlarged inset that shows the region-of-interest (0−4.5 μm). The characteristics of ETL lens at varying voltage or current is shown in Fig. 3c. Figure 3a shows the schematic for calibrating scanSMLM system using fluorescent nano-beads (size ~170 nm, *ex*/*em*: 505 nm/515 nm, Invitrogen, USA). The beads were embedded in an agarose gel-matrix (see, supplementary figs. 8 and 9). Uniform distrubution of beads in the gel-matrix is ensured. A 3D block of the gel-matrix is cut and imaged using scanSMLM (cyclic scan). The images were recorded and the corresponding raw data is shown in Supplementary Video 1 and 2. For reliable periodic scanning of specimen layers, we have correlated each plane with itself after a complete periodic scan i.e, between plane *i* and *i* + 10. The corresponding correlation plot is shown in Fig. 3d along with the Supplementary Video 1. A small variation in correlation ascertains the reliability of the system for cyclic or periodic scanning scheme. In addition, we have carried out drift measurement over the data collection time as shown in Fig. 3e. We noted a z-drift of ~ 0.2 nm/min for the reported data. Along with other chosen parameters (z-sampling, region of interest and the distance from coverslip), the system is then employed for data collection. Details of the experiments for determining scanning range, and drift-correction is detailed in Supplementary note 3 and Supplementary Video 1.

### 3D Imaging of actin filaments and mitochondrial network in NIH3T3 cells

**Fig. 4:**
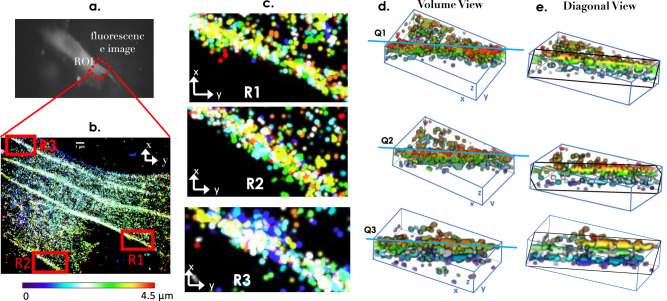
Arrangement of Actin filaments in a cell volume. **a** Fluorescence image of a typical NIH3T3 cells transfected with Dendra2-Actin plasmid DNA. **b** A small section of the cell is imaged using scanSMLM and 3D super-resolved volume is reconstructed, where colormap represents the depth. **c** Few 3D sections are displayed that show Actin filaments at different depths in a single cell. **d**, **e** Volume views along with the diagonal views (along lines Q1, Q2 and Q3) showing the arrangement of single Dendra2-Actin molecules. Scale = 1 μm. Additional details can be found in Supplementary note 4.

The system (scanSMLM) is used to visualize the 3D arrangement of Actin-filaments in a NIH3T3 cell as shown in Fig. 4a, b. F-actin to-gather with other cytoskeletal polymer controls the cellular shape and mechanical properties. High concentrations of crosslinkers that bind to actin filaments promotes the assembly of highly organized, stiff structures and forms aligned bundles that support filopodial protrusions, which are involved in chemotaxis (directed movement along a chemical gradient) and cell-cell communication. Photoconvertible fusion of fluorescent proteins Dendra2-Actin-C-18 (Addgene 57701) is used to visualized actin molecules in 3D. Figure 4c show the scanSMLM super resolves 3D image of bundles of actin upto a depth of 4.5 μm, each slice separated by 500nm. Area of dense(bundle) and spares of Actin molecule can be seen. Different sizes of bundles of actin are observed throughout the cell volume, ranging from 200 nm to 1 μm in lateral, whereas 500 nm to 1.5 μm in the axial direction. The size is independently confirmed by confocal studies that show an average span of 440 nm and 1.35 μm in the lateral and axial direction, respectively. More details can be found in Supplementary note 9, and supplementary figs. 10 and 20. Molecules in the bundles are found to tightly packed throughout the planes. The fluorescence image of a transfected cell along with the ROI is shown in Fig. 4a. The transfection is carried out by Dendra2-Actin plasmid DNA using the standard protocol as discussed in methods section. The experiment (recording of single molecules) is carried out to capture 10 different layers of cell (top-to-bottom), and the super-resolution volume map is reconstructed (see, methods section C for cyclic reconstruction protocol). Three regions (R1, R2, and R3) of Actin-filaments are chosen for 3D visualization as shown in Fig. 4d, e. In addition, volume view and corresponding diagonal views (along Q1, Q2, and Q3) are displayed to visualize the 3D arrangement of single molecules in Actin filaments. The corresponding reconstructed images and 3D volume map are shown in Supplementary note 4 and Supplementary video 3, respectively.

**Fig. 5:**
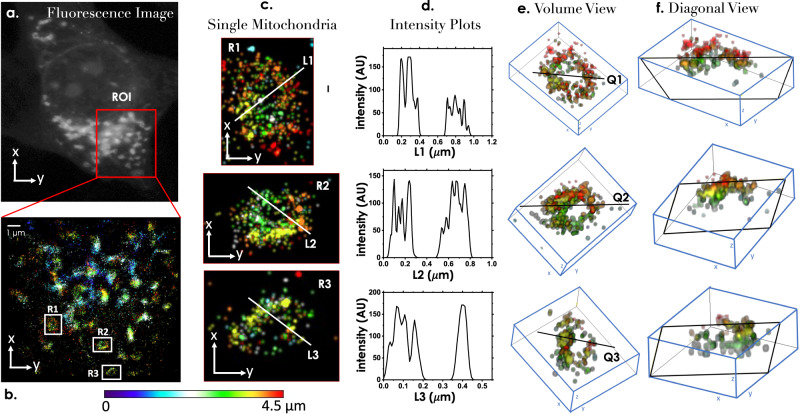
Distribution of mitochondrial network in a cell volume. **a** A high-resolution fluorescence image of mitochondria in a NIH3T3 cell (transfected with mEos-Tom20 plasmid DNA). **b** A section of the mitochondrial network is super-resolved and the volume is reconstructed. The depth is represented by colormap (see colorbar scale). **c** Few chosen single mitochondria (R1, R2, R3) are resolved that shows the distribution of single molecules (Meos-Tom20). **d** The corresponding line intensity plots suggests the distribution of single molecules on mitochondria (L1, L2, L3). **e**, **f** Volume views and the corresponding diagonal views (along lines, Q1, Q2, Q3) display the organization of single molecules across cell depths in a single mitochondria. Additional details can be found in Supplementary note 5.

In another experiment, we targeted mitochondrial network in NIH3T3 cells (see Fig. 5a). Cells were transfected with mEos-Tom20 plasmid DNA (mEos3.2-Tom20-N-10 Addgene, 57483) as per the protocol developed (see “Methods” section). Volume data (cyclic scan scheme) were recorded and processed as per reconstruction protocol (see “Methods” section). The corresponding volume image is shown in Fig. 5b, where the colormap represents different layers of cell volume. Alongside enlarged view and intensity profiles along specific lines (L1, L2, L3) are also shown in Fig. 5c, d. Volume of single mitochondria and the corresponding diagonal views are also shown that displays the organization of single molecules on plasma-membrane of mitochondria (see Fig. 5e and f). Overall, both the examples demonstrate the ability of scanSMLM for superresolved volume imaging. Tom20 is central component of the TOM (translocase of the outer membrane) receptor complex that is responsible for the reorganization of translocation of cytosolically synthesized mitochondrial proteins. Within a depth of 4.5 μm, total 10 planes are acquired with a step of 500 nm. Subsequently super-resolved image is reconstructed. All planes are stack together and hence 3D image is obtained. Previous studies reveal that the mitochondrial outer membrane structure is found to be thin envelope that encloses hollow space and of different shapes and size^22,55^. Similar structure is observed in 4.5 μm range. Mean localization precision of the TOM20 molecules is found to be 41.3 nm. Additional super-resolution images and 3D map are presented in Supplementary note 5, supplementary fig. 11 and Supplementary video 4, respectively.

### Organization of HA molecules in transfected cells

**Fig. 6:**
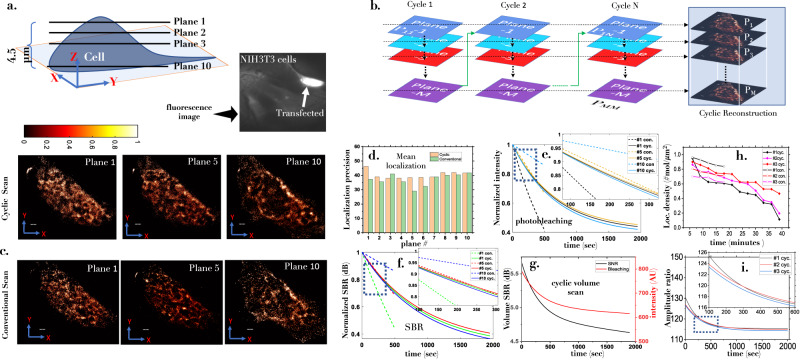
HA molecules in a cell volume. **a** A cartoon of 3D cell along with real fluorescence image of the transfected NIH3T3 cell. **b** Cyclic scanning for entire cell volume along with reconstructed super-resolved images (*P*_1_ to *P*_*M*_). **c** Super-resolved images of a few sample planes (plane 1, plane 5, and plane 10) reconstructed from raw recorded data acquired using cyclic and conventional scanning scheme. **d** The corresponding mean localization precision of all the reconstructed images (plane 1–10). **e** Plane-wise photobleaching study showing the decrease of fluorescence with time for both cyclic and conventional scan. **f** Signal-to-background ratio (SBR) for cyclic and conventional scan. **g** Change in SBR during cyclic scan. **h** The change in localization density with time. **i** Amplitude ratio versus time for cyclic scan. Scale = 1 μm. Additional details can be found in Supplementary note 6.

Next, scanSMLM system is employed to understand and quantify HA clustering in a Dendra2-HA transfected (Influenza A model). The transfected cells were identified using a blue light (470-490 nm) and the data is recorded by a sensitive EMCCD camera. The ETL is synchronized with the camera which is operating at 16*frames*/*s*. Each cycle consists of 10 images collected from respective Z-planes (plane 1-10) of a single cell as shown in Fig. 6a. Many such cycles are taken (see, Fig. 6b). Other details related to frame rate, number of planes, and number of cycles can be found in the methods section. Technically, single molecule events can be quantified (its position and size) directly in a 3D volume, and the distribution of HA molecules can be obtained in a 3D cell volume. We have employed both conventional and cyclic scan for volume reconstruction. Here, conventional scan necessitates data collection from cell planes in a sequential manner i.e, performing standard SMLM on each plane. Few selected 2D reconstructed planes (*#*1, *#*5, *#*10) within the cell volume are shown in Fig. 6c. More details related to other planes, sample raw data (cyclic scan) and sample raw data (conventional scan) can be found in Supplementary note 6, Supplementary figs. 12–15, and Supplementary Video 5 and 6, respectively. The volume view of 3D clusters of Dendra2-HA molecules are displayed in Supplementary Video 7. The average localization precision for both the scanning schemes are shown in Fig. 6d. This suggests that there is no observable particle resolution shift (PAR-shift) for planes at varying depths^56^. In addition, photobleaching characteristics for both cyclic and conventional scan are displayed in Fig. 6e, g. It is evident that cyclic scan show a steady decrease in photobleaching whereas it varies for conventional scan depending upon the z-plane. The corresponding signal-to-background ratio (SBR) follows a similar trend as shown in adjoining plot (Fig. 6f). This is predominantly due to exposure of a other planes when data is acquired for a specific plane. To determine the number statistics of single molecules over time, we have plotted localization density with time (for both cyclic and conventional scanning schemes), as shown in Fig. 6h. In addition, we have plotted amplitude ratio (defined as the ratio of amplitude and offset of the fitted PSFs) versus time in Fig. 6i. These metric indicates a steady decrease in the number of single molecules with time for conventional compared to the cyclic scheme, suggesting that the cyclic scheme is better suited for long-term imaging. Overall, the advantage of cyclic scan over conventional scan is evident in terms of SBR and overall photobleaching.

### Volume visualization of HA clusters and biophysical parameter estimation

**Fig. 7:**
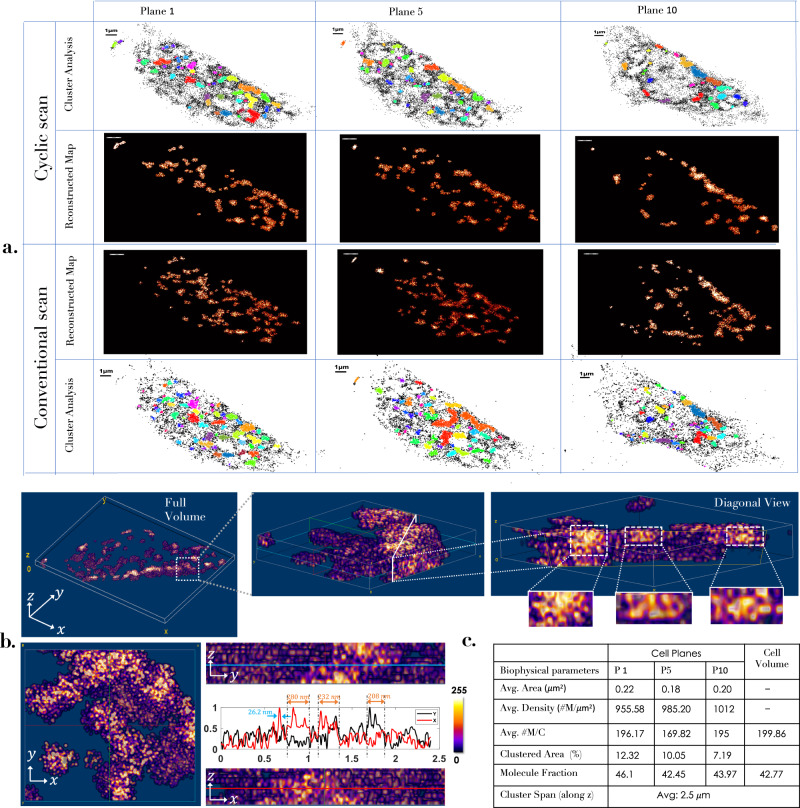
Clustering of HA molecules in a cell volume. **a** Cluster map of the distributed HA molecules in a transfected NIH3T3 cell (of few chosen planes at 500 nm, 2500 nm and 4500 nm depth) for both cyclic and conventional scanning. DBSCAN algorithm is used to analyze clusters (with parameters radius *ϵ* = 114 nm and minimum molecules of 35 for it to be designated as a cluster). Analysis show aggregated HA molecules throughout the cell volume. **b** Volume image of a cell displaying 3D distribution of HA molecules. In addition, diagonal sectional view of the 3D cluster indicates the local density of clusters across the cell volume. Few enlarged section of a single cluster are also displayed along with orthogonal sectional views (along XY, YZ, XZ) of a specific region. Corresponding intensity plots indicate average HA cluster size of ≈ 240 nm (see, orange arrow) and typical single molecule size (localization precision) of 26.2 nm (see, blue arrow). **c** Estimated biophysical parameters (average cluster area, density and *#* molecules per cluster) related to local cell physiology indicates that most of the clusters are spread over a distance of 200 nm with an average density of about 1000#mol./μm^2^. In addition, estimated from the cell volume shows the clustered molecule fraction of ~42.77, indicating that 42.77% of the total HA molecules are clustered, and rest remains unclustered, post 24 hrs of transfection. The clustered area fraction (which is the ratio of clustered area to the total cell area) is found be about 0.981%, indicating a < 1% area is occupied by clusters. These parameters are critical for understanding HA dynamics and help determine underlying biophysical processes. More details can be found in Supplementary note 7 and Supplementary note 8.

To determine critical biophysical parameters, we have employed both conventional and cyclic scanning scheme for which the reconstructed images are as shown in Fig. 7a. The images are then subjected to cluster analysis. DBSCAN algorithm is used to identify the clusters and determine the parameters. Post 24 hours of transfection, the HA molecules are found to form clusters. We identified ~45 clusters in a single plane. To better visualize the organization and close packing of HA molecules, we have shown reconstructed volume map in Fig. 7b. More details related to cluster in 3D are shown in Supplementary note 7. It appears that the clusters are well connected across the layers of 3D cell. This is better visualized in the diagonal view and the related enlarged sections. In addition, orthogonal sectional views (XY, XZ, YZ) are also shown. Note that, the algorithm removes unclustered molecules that facilitates study of only the clustered molecules. A majority of the cluster size range from 0.18 − 0.22 μm^2^ with an average cluster size of 0.2 μm^2^. The cluster density is known to be a critical indicator for accessing the progression of viral infection. The average density is found to lie between 950 − 1050*#*M/μm^2^. Surprisingly, the number of HA molecules per cluster varied from 160 to 200. These critical parameters are tabulated in the Fig. 7c for few chosen planes (*P*1, *P*5, *P*10) of the transfected cell. Analysis on the entire cell volume indicate that a clustered molecule fraction (ratio of clustered to total HA molecules in the cell volume) of 43%. Another useful indicator is the cluster area fraction (ratio of the area occupied by the clustered molecules to the total area in a planes) which is found to be 9.85% per plane (average value). In addition, we have also observed a significant variation in the number of molecules per cluster per plane, while it is ~200 molecules per volume. Another interesting parameter is associated with the span of clusters in the cell volume. It is observed that most of the cluster span over large extent post 24 hrs of transfection. Specifically, 82% of the clusters span for more than ≥2 planes (~1 μm), and an average of 5 clusters span over ~2.5 μm planes which is approximately half the cell volume. The corresponding details are discussed in Supplementary note 7 and supplementary figs. 16–18. In addition, we have carried out confocal studies that confirm connected clusters throughout the cell volume. The size of the cluster range from 1.08 to 1.62 μm along the *z*-axis (axially). More details can be found in Supplementary note 8 and supplementary fig. 19. This clearly demonstrates that clusters connect across the cell volume which is not revealed by the existing SMLM techniques. This has consequence on the rate of infection. The above parameters gives critical information related to rate of infection in a cell volume^57^. We anticipate that volume (considering all the planes) based biophysical parameter estimation facilitated by scanSMLM may give a better understanding of HA clustering during Influenza-A viral infection.

## Discussion

A scanning super-resolution volume imaging system (scanSMLM) is developed. Unlike existing SMLM techniques that are capable of reconstructing a single plane, the proposed system can reconstruct the entire volume. The newly developed cyclic scanning periodically scans and records single molecule signatures from all the planes of a target volume in an unbiased manner, and directly reconstructs the super-resolution volume map. The correlation study shows high repeatability (>94%) of the planes for long imaging cycles. In practice, this is achieved by using an electrically-tunable lens for focusing fluorescence from respective z-planes onto the detector. It is evident that ETL has the dual advantage of focal position control and high-speed manoeuvring of the focal position. The fast response time of ETL (~5 ms) in principle allows a scan speed of ~200 Hz. The technique has the added advantage of rapid axial scanning in a closed system (such as high-resolution endomicroscopy) that necessitates motion-free mechanical scanning. Other advantages of scanSMLM (cyclic) are bias-free scanning of *z*-planes, motion-less *z*-scanning, elimination of repositioning error (due to bulky objective or stage movement), avoiding bulky cumbersome systems (objective with piezo stage), and order faster scanning. These features pave the way for the miniaturized realization of high-resolution super-resolution microscopy.

For high-quality imaging, the system requires precision calibration and testing on a known specimen. In the 4*f* detection sub-system, the ratio of the focal length of the objective lens to tube lens (ETL) is very high (1:4000). This facilitates fine *z*-scanning of the object as a function of ETL focal length which can be precisely controlled by finely varying the ETL current. The auto-tunable lens offers flexible and high-precision scanning which is largely due to its sensitivity (smallest change in current is 0.07mA) that allows in principle z-shift (in the object plane) measurement of 17.5 nm (for the present scanSMLM system). Another special feature of ETL is its ability to retract to its original position, thereby minimizing repositioning error (see, correlation function in Fig. 3). This feature allows reliable scanning of closely-spaced z-planes in the target volume. To demonstrate precision z-sectioning capability, we have imaged fluorescent beads (size ~ 175 nm). While scanSMLM is impressive and quite accurate, we did observe *z*-drift at a rate of ~ 0.2 nm/min. Though the rate is negligible for standard data acquisition time, it is significant for hour-long imaging. It turns out that for our data collection of 50 min, we observed a total z-shift of ~11 nm. Accordingly, all the data is corrected for drift.

Here, the scanSMLM is used to image fine structures in the cellular system. To demonstrate, the system is used to image actin-filaments and mitochondrial network (see Figs. 4 and 5). We have used photoactivable/photoconvertible proteins as compared to photoswitchable dyes to ensure bio-compatibility and non-toxicity. Since proteins get expressed in the cell they are non-toxic and offer live cell study as well. Using scanSMLM, we could visualize the arrangement of Dendra2-Actin molecules on actin-filaments in a 3D cell that gave a plethora of information about its positioning, and density across the cell volume. A similar experiment is performed to visualize the mitochondrial network in a cellular system and the organization of single molecules (mEos-Tom20) on a single mitochondria (see, Fig. 5). Results show the distribution of the single molecules on the surface (outer membrane) of mitochondria. This demonstrates the ability of scanSMLM for visualizing minuscule three-dimensional structures across the cell volume and even allows probing these fascinating organelles at an individual level.

To access the resolution of the reconstructed images, we have carried out Fourier Ring Correlation (FRC)^58–62^. Compared to other techniques, FRC does not require any prior information, and the metric can be computed in real-time to determine image resolution. FRC is based on the fact that every system has an effective cutoff frequency, and the resolution is determined by high spatial frequencies supported by the system. Here, we employed FRC metric to compute resolution for two different specimens, i.e., cells transfected with Dendra2-Actin and Dendra2-HA. Analysis shows a resolution of ~142 nm for Actin filaments, whereas it is 165.40 nm (cyclic scheme) and 163.81 nm (conventional scheme) for HA clusters in NIH3T3 cells. A detailed analysis for both cases can be found in Supplementary note 10 and supplementary fig. 21.

Finally, to demonstrate the benefits of scanSMLM technique in disease biology, we used it to understand the 3D organization of HA protein and its aggregation in a Dendra2-HA transfected NIH3T3 cells (Influenza A model). scanSMLM provided the first 3D image of these aggregates/clusters in a cell volume (see Fig. 7). Immediately, we could visualize clusters that are connected across the cell volume (post 24 h of transfection) and are not just limited to a specific plane/region as reported using standard single-molecule SMLM techniques. However, we did observe a slight change in localization precision whereas, other parameters related to imaging (such as photobleaching and SBR) are found to be as expected in standard SMLM. In principle, one can target a potential drug that may inhibit clustering across the planes in a cell volume and promote HA declustering, a potential step toward drug-discovery. We have largely used cyclic scan for our study which is largely due to its uniform and unbiased data collection throughout the cell volume. Finally, cluster analysis is performed to determine biophysical parameters both at the level of a single plane and the entire volume.

In our study, we have chosen to work with DBSCAN clustering algorithm. Another well-known and efficient clustering algorithm is point-clustering^63^. We have discussed and shown the results for point clustering in Supplementary note 7. It may be noted that scanSMLM enables visualization of single molecules clusters beyond a single plane, and thus can decipher fine details across the planes in a 3D cell. Further analysis is carried out to determine the biophysical parameters (such as average cluster size ~240 nm, the average number of molecules per cluster, ~200, fraction of clustered HA molecules ~43% and its span (average span of a cluster ~2500 nm) in the cell volume, apart from other parameters) that are directly linked to infection rate. We anticipate that scanSMLM can be used to investigate other fundamental problems (in clinical biology and applied physics) that are plagued by limited understanding offered by existing SMLM techniques.

## Methods

### System design

We customized the traditional epifluorescence mode SMLM to implement scanSMLM (see, Fig. 1). Similar to conventional SMLM, the activation laser(405nm) and readout laser(561nm) are combined with an appropriate dichroic mirror. The combined beam is then directed to the 100X, 1.3NA objective lens of Olympus IX81 inverted microscope. An additional ETL is introduced in the beam path to set desired illumination field-of-view. The fluorescence from the specimen is separated from illumination light by a dichroic mirror of cut-off 561 nm (installed in the filter cube of the microscope) in addition to notch filters (405 nm and 561 nm notch filters) and focused by tube lens of invented microscope at the primary image plane. The primary image is magnified by 2.8X using a combination of ETL(*f* = 350 mm) and L2 (*f* = 125 mm). Hence, the detection subsystem system has a total magnification 280 (100 × 2.8). We used an Andor EMCCD camera with a physical pixel size of 16.5 μm to record the single-molecule data. With a magnification of 280X in the detection sub system, the pixel size (q) is approximately 59 nm. Different planes of the specimen were recorded by tuning the focal length of the ETL. The focal length was altered by sending external voltage to ETL 4i driver. The calibration of the selectivity of focal length and corresponding z-position in the sample is carried out using nano-beads and with known microscope mechanical z-stage knob labels (as per manufacturer’s specification). Two schemes were adopted: cyclic and conventional. Single molecule data were acquired, and 5000 cycles per plane of the specimen were recorded for reconstruction. The user can set any arbitrary voltage (in the range 0—5V) to select a specific scanning plane.

### Electrically tunable lens and automation

Selectivity of the z planes is achieved by tuning the curvature of the ETL. Mechanically, ETL consist of a container filled with optofluids and is sealed by elastic polymer membrane. The membrane deflection is proportional to pressure in the fluid. The pressure in fluid container is applied through electromagnetic actuator. Therefore, current flowing through electromagnetic actuator is responsible for change in focal length. User controlled external voltage applied to 4i driver can be used to drive current flowing through the actuator. In scanSMLM, ETL is used to scan the specimen.

The ETL (EL − 10 − 30 − CI − VIS − LD − MV) used in this work is purchased from Edmund Optics, Singapore. Electrically tunable lenses, also known as liquid lenses, can change their curvature rapidly upon application of current to its actuator. The key element in ETL is a container filled with optical fluids, which is sealed off with a thin elastic polymer membrane. A current-controlled actuator pushes the liquid into the center of the membrane and deflects it. As a result, the radius of the container can have different configurations (from concave to convex). The current in lens actuator is controlled by a 4*i* lens driver. This lens drive has a control from −290 mA (concave) to +290 mA (convex) with 12 bit precision and has 0.07 mA incremental steps response. An external analog input pin that accepts 0−5V is used as input to the driver to drive the set current range in ETL.

Specifically, two scheme are employed: cyclic and conventional. The external applied voltage (0–5V) is generated using digital potentiometer which is digitally programmed via Arduino. In practice, the smallest change in ETL-current is 0.07 mA and the total range is 0−290 mA. In our implementation, we used a range of 200−218 mA with a change of 2 mA. This corresponds to a change of focus 350−370 mm at a step size of 2 mm, which is equivalent to a range of 5 μm and inter-plane spacing of 500 nm in the object plane. Accordingly, the developed system was optimized for a range of 5 μm along z-axis with a sampling of ~0.5 μm.

### Algorithm and coding for ETL

Arduino script is developed to generate driving voltage with a range of 0−5V and a voltage-step of 0.5V. This is executed by an external digital potentiometer (10*kOhm* with 256 available steps). The interfacing of Arduino board with potentiometer is achieved by serial communication protocol (SPI). The flow-chart of controlled program sequence and related details are discussed in Supplementary note 2. The entire code and related details can be found in GITHUB (https://github.com/jigmibasumatary/scanSMLM.git).

### Cell culture and transfection method

NIH3T3 mouse fibroblast cell line is a generous gift from Prof Deepak Nair (Centre for Neuroscience, Indian Institute of Science, Bangalore, India). To begin with, the cells were thawn from preserved specimen in liquid Nitrogen container LN2 cryocan (−196 °C). The NIH3T3 cells are maintained in Dulbecco’s Modified Eagle Medium (DMEM) complete media (89% of DMEM medium (Gibco, Thermofisher, USA), 10% Fetal bovine serum (FBS) and 1% Penecilin-Streptomycin (Gibco, Therofisher, USA)) at 37^∘^C and 5% CO2 (CO2 -incubator, Thermo Scientific, USA). For Dendra2HA plasmid transfection, NIH3T3 cells were seeded at 10^5^/mL concentration on a cover slip(No. 0, thickness 0.1 mm) (Bluestar, India) in a 35mm petridish (Biofil, India) or on a live imaging dish (MatTek, USA) and were cultured for 12h. On the day of transfection, the cells were transferred to fresh DMEM (serum-free) media and transfected with Dendra2HA plasmid (gift) using lipofectamine 3000 (Life Technologies, Invitrogen, USA) following standard manufacturer’s protocol (Invitrogen, USA) and incubated for 24 h. The Dendra2HA transfected cells were then washed thrice gently with warm PBS (Gibco, Thermifisher, USA), followed by fixation with 3.7% PFA at room temperature for 15 min. Subsequently, PFA was removed by washed thrice with PBS and mounted on a glass-slide using Fluorosave mounting media (Invitrogen, Carlsbad, CA, USA) to keep cells in a hydrated form for a long duration.

### Dendra2-Actin

Actin filaments forms the cytoskeleton. It is made up of linear polymers of globular actin subunits and forms microfilaments which in turn make linear bundles. The fluorescent protein Dendra2 is N-terminally tagged with Actin and named as Dendra2-Actin (It was a gift from Samuel Hess,NIH). Cells were transfected with Dendra2-Actin plasmid using Lipofectamine 3000(Life Technologies, Invitrogen) according to manufacturer’s protocol. Post transfection, the cells were incubated for 24 hrs and checked for protein (Dendra2-Actin) expression. The expression is confirmed by visualizing it in a epifluorescence microscope (IX81 Olympus Inc., Japan) with a blue light (470 − 490 nm) illumination that gives fluorescence at 507 nm. However, in the super-resolution mode, the photoactivable characteristics of protein is exploited by activation it with a 405 nm light followed by excitation at 561 nm for which the emission occurs at 573 nm (emission peak).

### mEos-Tom20

Tom20 is a mitochondrial protein, a translocase of the outer mitochondrial membrane, which is responsible for the translocation of cytosolically synthesized mitochondrial- targeted proteins. TOM20 is C terminally tagged with fluorescent protein mEos. mEos is a photoconvertible protein, directly excitable by blue light (470 − 490 nm) with fluorescence emission at 516 nm. Cells transfected by mEos-Tom20 plasmid were visualized using blue light. To obtain super-resolution data, photoconvertable property (activation and excitation by 405 nm and 561 nm light with its emission at 580 nm) of the probe is used.

### Dendra2-HA

Haemagglutinin (HA) is glycoprotein found on the envelope of influenza virus that help its entry to the host cell. HA is known to form clusters in the cell membrane which is essential for replication. The fluorescent protein Dendra2 is N-terminally tagged with HA and hence named as Dendra2-HA (It was a gift from Samuel Hess,NIH). Dendra2-HA enters the cell using lipid-mediated transfection and gets expressed. The expression of the protein is visualized by exciting the fluorophore (Dendra2). Dendra2-HA is a photoactivable protein, that gives fluorescence at 507 nm when exposed to UV (405nm), and also gives fluorescence at 572 nm when excited by 561 nm.

### Data acquisition and image processing

For data acquisition, the ETL is synchronized with the camera, which is operating at 16*frames*/*s*. Each cycle consists of 10 images collected from respective Z-planes (plane 1-10) of a single cell with an inter-plane spacing of 500 nm as shown in Fig. 6a. Many such cycles are taken to obtain enough number of single molecule signatures throughout the volume (see, Fig. 6b). This sums up to a total of *M* × *N* images. For our study, we have taken *N* = 5000 cycles, and *M* = 10 planes. The same process is carried out for all the cell experiments.

Conventional Gaussian fitting approach is used to localized molecules. Each 16-bit .tiff frame is initially converted into photon count using the pixel-to-photon ratio. The photon to pixel ratio is the measure of the slope of variance vs. mean intensity^25^. Single-molecule reconstruction of the single molecules is done using modified Einzel code on Matlab2021b. The reconstruction follows the standard steps. The first step of the process is background subtraction using rolling ball algorithm^64^. Following background subtraction, identification of bright spots (representing probable molecule) is carried out by thresholding intensity. All the pixel values above the cutoff threshold (T1) is tabulated. A window between 3 × 3 pixels and 7 × 7 pixels is considered for identifying single molecules and for determining its centroid and number of photons. The centroid calculation gives the initial guess coordinates for Gaussian fitting. The amplitude of the Gaussian fit gives the number of photons detected from that molecule. The localization of the single molecules is calculated Thomson et al.^25^. Each fitted molecule is rendered by weighted plots of the position. The localized molecules are then plotted as a spot having a Gaussian profile with intensity proportional to the total number of photons detected on it and radius equal to the experimentally calculated localization-based resolution. The weighted render technique accounts intensity and position of each molecule and is a more realistic means for representing the molecules.

The acquired tif images are processed plane-wise for image reconstruction. Initially, 16 bit grayscale image is converted into photon count by multiplying pixel-to-photon ratio of the EMCCD. Subsequently background noise is subtracted using the rolling ball algorithm^64,65^. This method allows background subtraction in each frame independently. Briefly background in each image is generated by first smoothed (convolution with a two-dimensional Gaussian distribution) and then dilated with a rolling ball. The generated background profile is subtracted from raw images for further processing. The radius of the circle (rolling-ball radius) is chosen to be larger than PSF size. The spots in background-free images are identified by thresholding intensity. The threshold values are chosen so that the algorithm does not accidentally select noise as molecules. To localize each detected molecules, 2D Gaussian is fitted with an appropriate initial guess to reduce the fitting time. The output parameters of the Gaussian fit are position coordinates and amplitude. Using fitting parameters, the number of photons together with noise and effective pixel size, Thomson’s localization formula is used to calculate the localization precision. Furthermore, each molecule is rendered using weighted 2D Gaussian. In the present technique, we have used a rolling ball radius and detection threshold of 347 nm and 23 photons, respectively.

### Drift correction and analysis

Typical drift characteristics of the scanSMLM is characterized using immobile 170nm green fluorescence beads. A cyclic volume data of 100 planes of the immobile beads is performed after every 1 minutes. From the acquired data lateral drift (xy-drift) is observed at a specific plane as a function of time. The distance shift between initial localized centroid to final localized centroid represents amount of drift. Axial drift(z-drift) is calculated by noting down the position shift of the highly focused plane of the immobile bead(50th plane) to the subsequent cyclic volume scan. Highly focused plane and corresponding position of the immobile bead in the subsequent scanning is identified by highest cross-correlation value^66,67^. The amount of position between initial and final time is axial drift. Figure 3E shows the drift characteristics for the developed scanSMLM system. Drift is observed over 50 minutes, and an axial drift rate of 0.20nm/minutes is noted. All the reconstructed images are corrected for drift.

### DBSCAN Clustering

Once inside the cellular system, HA molecules are known to cluster (post transfection)^49,68^. We employed DBSCAN algorithm to identify and analyze clusters. DBSCAN allows the determination of critical biophysical properties such as area, *#* molecules per cluster, density (*#mols*. /*area*), molecule cluster fraction and area fraction. These parameters are indicators of infectivity.

DBSCAN algorithm works by identifying the molecules that belong to a specific cluster. Specifically, DBSCAN uses two parameters: Distance measure that can be used to locate points in the neighborhood of any point, and minimum number of points (a threshold) for recognizing a assembly of HA molecules for it to be considered as a cluster. The choice of distance function makes a big impact on the outcome. For our case, we used Euclidean distance measure (distance between two points, (*x*_*i*_, *y*_*i*_) and (*x*_*j*_, *y*_*j*_)) given by, $d={\sum }_{i,j}\sqrt{{({x}_{i}-{x}_{j})}^{2}+{({y}_{i}-{y}_{j})}^{2}}$. The DBSCAN algorithm has the following key steps:

1. The algorithm begins with any point in the set, and explores all the points.
2. Within the radius of the point, other points are located and if the number of points are at least the specified minimum point, then all these points together constitute a cluster.
3. The clusters are then recursively expanded by visiting neighborhood points.

The algorithm visit all the points, and the number of clusters are identified.

Other competitive clustering techniques are, K-means, PAM clustering, and hierarchical clustering that works better for convex or spherical clusters in the data. These techniques are better suited for compact and well-separated clusters but fail miserably in the presence of noise. Our study (HA distribution in a transfected cell) calls for techniques that are robust in the presence of noise and are able to identify arbitrary-shaped clusters that are well-known in cellular system. The advantage of DBSCAN over other clustering techniques is its ability to identify clusters having arbitrary shapes, reduce noise and are able to discard points that do not belong to any cluster. This is beneficial for disease biology (e.g., Influenza-A) since clustered molecules has a direct bearing on the rate of infection. Finally, all the clusters are taken into account and further analysis is carried out to determine critical biophysical parameters (see, Fig. 7c).

### Signal to background ratio

SBR is an important indicator for determining signal strength with respect to the background. Hence, it is a measure that directly relates to the quality of reconstruction in super-resolution microscopy. Mathematically, SBR is defined as the ratio of average (*μ*) to the standard deviation (*σ*) of images i.e,

1 $$SBR=10\times {\log }_{10}\,(\mu /\sigma )$$

### Normalization parameter

The images are normalized prior to analysis. Normalization is carried out in the range [0,1] using the following standard formula:

2 $$Normalized\,Intensity=\frac{I(x,y)-\min [(x,y)]}{\max [I(x,y)]-\min [I(x,y)]}$$

where, $\min [I(x,y)]$ and $\max [I(x,y)]$ are the minimum and maximum values, respectively.

### Reporting summary

Further information on research design is available in the Nature Portfolio Reporting Summary linked to this article.

### Supplementary information

Supplementary Material

Description of Additional Supplementary Files

Supplementary Data 1

Supplementary Video 1

Supplementary Video 2

Supplementary Video 3

Supplementary Video 4

Supplementary Video 5

Supplementary Video 6

Supplementary Video 7

Reporting Summary

## Data Availability

The data that support the findings of this study are available from the corresponding author upon request. Source data can be found in Supplementary Data 1.
