# Supplementary material for: Scanning single molecule localization microscopy (scanSMLM) for super-resolution volume imaging
Source: Commun Biol. 2023 Oct 17;6:1050. doi: 10.1038/s42003-023-05364-2 (PMC10582190; doi:10.1038/s42003-023-05364-2)
Supplement: Supplementary file 1 — Supplementary Material [file 42003_2023_5364_MOESM1_ESM.pdf]

Supplementary Material: Scanning Single Molecule Localization Microscopy (scanSMLM) for Super-resolution Volume Imaging

Jigmi Basumatary[1], Neptune Baro[1], Prakash Joshi[1] and Partha Pratim Mondal[1,2]
[1] Department of Instrumentation and Applied Physics, Indian Institute of Science, Bangalore 560012, INDIA
[2] Center for Cryogenic Technology, Indian Institute of Science, Bangalore 560012, INDIA

Supplementary Notes 1-11

Supplementary Note 1: Optical Setup of Actual *scanSMLM* microscopy system

Supplementary Note 2: Automation Circuit and Synchronization

Supplementary Note 3: Volume Scanning Calibration of scanSMLM on Test Sample (fluorescent nano-beads)

Supplementary Note 4: Cyclic Scanning of Actin Filaments in a transfected NIH3T3 cell

Supplementary Note 5: Cyclic Scanning of Mitochondrial network in a transfected NIH3T3 cell

Supplementary Note 6: Cyclic Scanning and Superseolution Volume Reconstruction of Dendra2-HA Distribution in a transfected NIH3T3 cell

Supplementary Note 7: Point-based Clustering of Dendra2-HA molecules in a cell Volume

Supplementary Note 8: Confocal Study of HA Clusters

Supplementary Note 9. Confocal Study of Actin Filaments

Supplementary Note 10. FRC Analysis

Supplementary Note 11. Noise Analysis

Supplementary Videos

Supplementary Video 1: Cyclic scanning of nano beads for calibration.

Supplementary Video 2: Cyclic scanning of micro beads (test sample).

Supplementary Video 3: Reconstructed 3D map of Dendra2-Actin molecules on Actin filaments.

Supplementary Video 4: Reconstructed 3D map of mEos-Tom20 molecules on the mitochondrial network.

Supplementary Video 5: Recorded super-resolution data (cyclic scan) of Dendra2-HA transfected cell.

Supplementary Video 6: Conventional scanning Dendra2-HA transfected cell.

Supplementary Video 7: 3D cluster of Dendra2-HA molecules.

Supplementary Note 1. Optical Setup of Actual *scanSMLM* microscopy system

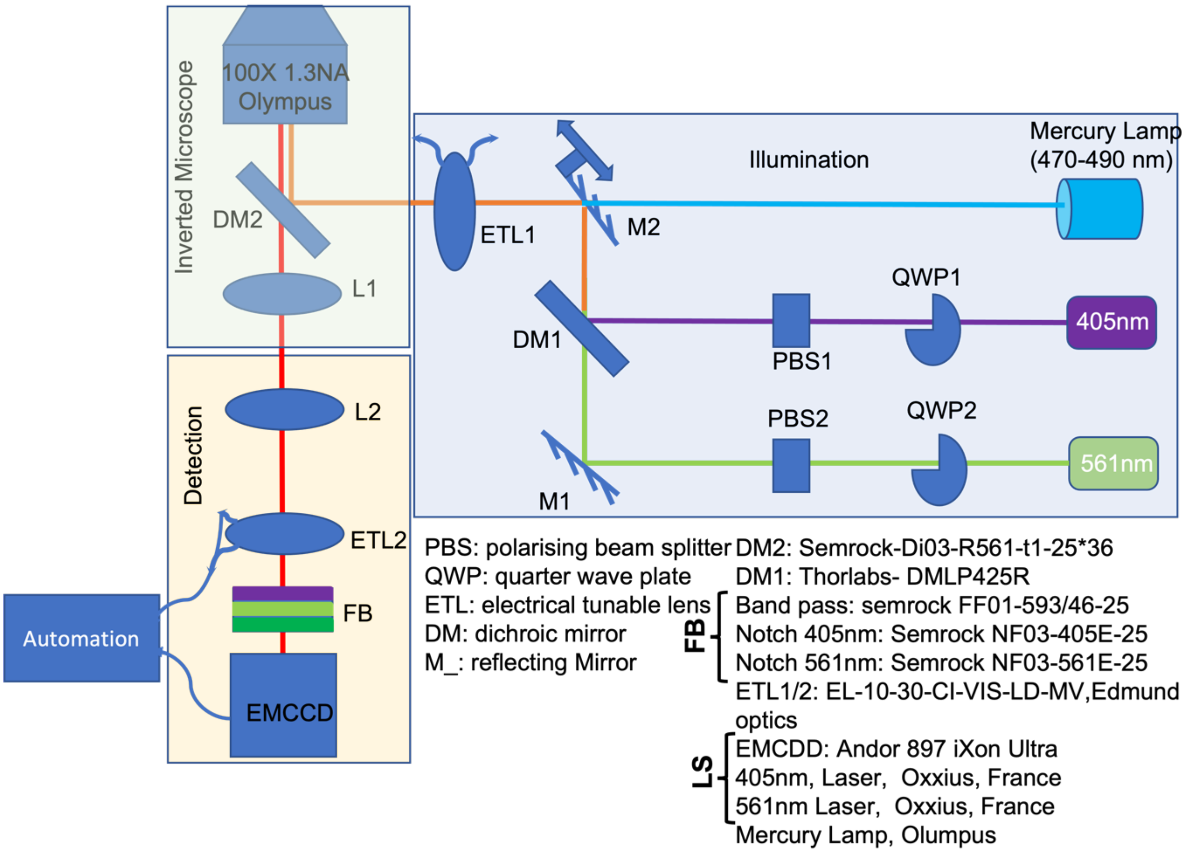

Supplementary Figure 1: Key optical components of scan-SMLM. Synchronizing ETL2 and EMCCD detector felicitates volume scanning (for both cyclic and conventional scanning scheme) of the specimen.

The scan-SMLM system is realised in a widefield mode (see, supplementary figure 1). The illumination sub-systems consist of 405 nm activation laser and 561 nm excitation laser. The intensity controller comprises of quarter wave plate and a polarising beam splitter which are introduced in each beam path. Both the beams are combined by a dichroic mirror (DM1) and directed to an electrical tunable lens ETL1. The ETL1 is introduced in the illumination path to set desire illumination field-of-view (FOV) [1]. The beam is then coupled to a high-resolution microscope (Inverted Microscopy IX81, Olympus Inc., Japan) equipped with high NA objective lens (100x 1.3NA Olympus) and a filter turret (containing dichroic mirror DM2 (Semrock, USA)). The Stoke-shifted fluorescence (peak at 573nm and 584nm for dendra2 and mEos3.2, respectively) from the specimen is collected by the same objective, transmitted by microscopic dichroic mirror (DM2) and focused by internal microscope lens (L1) to the side-port of microscope. Since, *scan-SMLM* system requires additional magnification, a series of magnification units are employed (combination of ETL2 and biconvex lens L2 (f=125 mm). The magnified image is then focused on to the EMCCD detector (Andor, Orford Instruments, UK). In addition, a set of filters (notch filters and bandpass filters) are used in the detection path to eliminate unwanted (illumination and stray) light. An additional optical set-up for blue light (470-490 nm) illumination is integrated with the microscope to visualize the transfected cell (emission peak of 507nm and 519 for dendra2 and mEos3.2, respectively). The actual *scan-SMLM* system that is used for data collection is shown in supplementary figure 2.

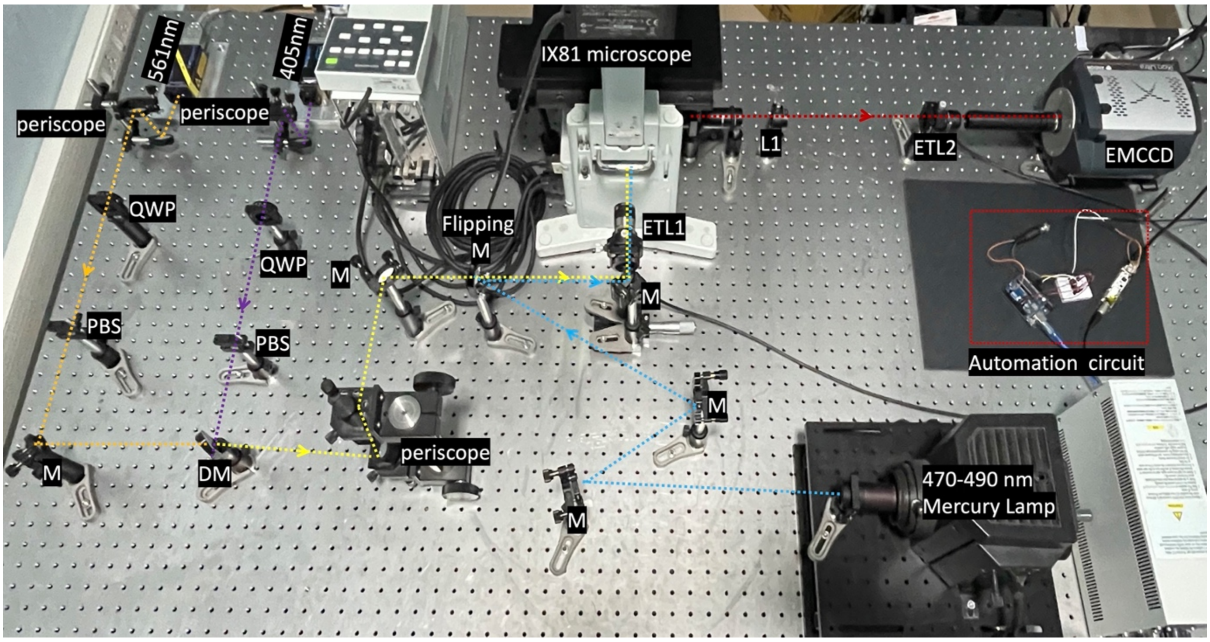

Supplementary Figure 2: Picture of the actual scan-SMLM super-resolution system. The system comprises of illumination and detection sub-system along with inverted fluorescence microscope and integrated fluorescence arm for blue-light illumination. Critical components (ETL lenses (ETL1 and ETL2), EMCCD detector, Automation circuit, Blue-light fluorescence arm etc.) are also indicated.

Supplementary Note 2. Automation Circuit and Synchronization

Automation circuit, ETL tuning and synchronisation

Automated cyclic scanning relies on the synchronization between ETL tuning and EMCCD data recording. The complete schematic of synchronized data collection module used in *scanSMLM* is shown in supplementary figure 3. To realize this, we controlled the ETL current set in the firmware software with driving voltage of 0-5V(DPM output. This alters ETL aperture thereby allowing fluorescence collection from respective cell layers (object plane) up to a depth of 4.5 μm. The corresponding images (containing single molecule signatures) are recorded and processed. The driving voltage is realized in discrete step generated by an external digital potentiometer which is fed to Pin-B of 4i driver of the ETL (opto-tunable lens in the detection). The step voltage is decided by the distance between the specimen z-planes, Δz=500 nm (object plane). The applied voltage (at pin B) is linearly mapped to the ETL operating current range defined by the manufacturer (lower and upper software limits in the hardware configuration tab of Lens Driver Controller). The current in 4i driver(200mA to 218mA) pilot the focal length (from 350mm to 370mm) of ETL. The interconnection functional tuning system is shown in supplementary figure 4

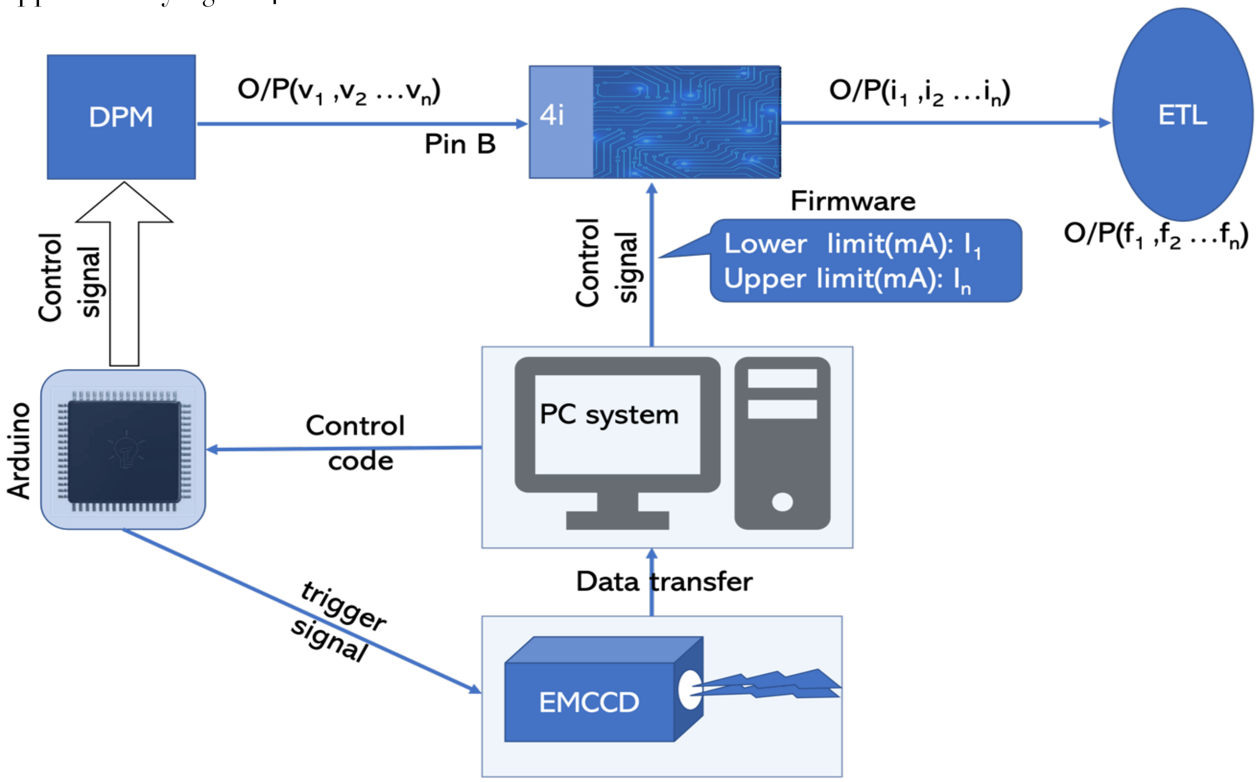

Supplementary Figure 3. The complete automation system comprising ETL, Arduino, DPM, ETL-driver, and the detector (EMCCD).

Driving voltage generation:

The digital potentiometer IC communicates with Arduino vis Serial Peripheral Interface (SPI) protocol. This potentiometer has a sensitivity of 8 bit and hence total 256 taps can be realized over 10 kΩs. It has typical wiper resistance of 125 Ωs. Theoretically, the terminal voltages are calculated as follows-

$$R_{WA} = R_{AB}\left(1 - \frac{D_n}{256}\right) + R_W$$
$$R_{WB} = R_{AB}\left(\frac{D_n}{256}\right) + R_W$$

where, D_n is a 8 bit value in the data register for pot #n, R_W is the wiper resistance, and R_{AB} is the total potentiometer resistance.

In Arduino code, we divided 10k resistance into 10 parts by setting n-steps, 25.6*n (where n =1,2,3….. 10 tap positions that correspond to plane number). Specifically, for our application, the current used in 4i-driver is

from 200mA to 218mA that correspond to focal lengths from 350mm to 370mm of ETL2. The interconnection functional tuning system is shown in supplementary figure 4.

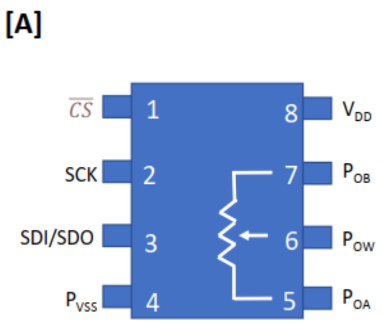

[B]

PIN #	PIN NAME	DESCRIPTION
1	$\overline{CS}$	Chip Select, pin is the SS (slave select) pin for the SPI interface. It is active low. 0V means the chip is selected and 5V means it is not selected.
2	SCK	SCLK is the Shared/Serial Clock. It is the SPI clock line.
3	SDI/SDO	Serial data in and out, also known as the MOSI and MISO.
4	P_{VSS}	Ground.
5	P_{OA}	Terminal A of the potentiometer.
6	P_{OW}	Wiper terminal
7	P_{OB}	Terminal B of the potentiometer.
8	V_{DD}	Positive voltage source.

Supplementary Figure 4. [A] PIN diagram of DPM (MCP41010). [B] PIN description of DPM.

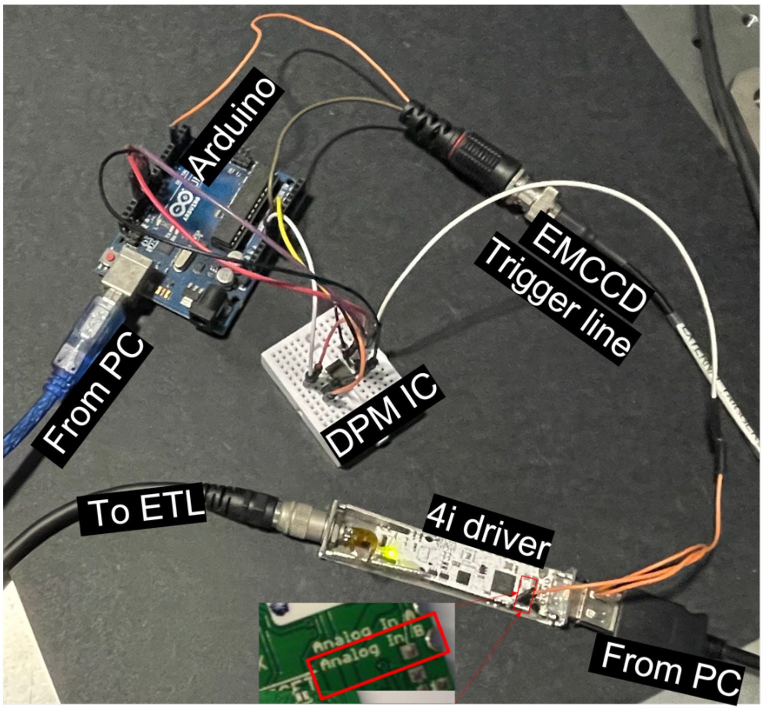

Supplementary Figure 5. Automation circuit and connections between Arduino, ETL Driver and the EMCCD camera..

The complete synchronization system is shown in Fig. S2-3. Specifically, the microcontroller, Arduino UNO R3 (arduino.cc) is used to synchronise the tuning of ETL and camera data acquisition (see, supplementary figure 5). The code flow chart for synchronisation and driving voltage for conventional and cyclic scanning scheme is shown in supplementary figure 6.

Cyclic Scanning: Control flow chart is depicted in supplementary figure 6. The acquisition rate in scan-SMLM is limited by time taken by ETL and camera. As specified by ETL manufacturer, the response time is $\sim 5\,ms$. The details timing diagram of both conventional and cyclic scan is already presented in figure 2 (main text). The actual time required by cyclic technique is given by,

$$t_{tot/cyc} = M \times \left(t_{exp} + t_r + t_d\right)$$

where, $M = 10$ is total #planes scanned in one cycle.

During imaging, several thousand cycles are repeated for acquiring data for reconstructing super-resolved image. For, $N=5000$ cycle, the time taken for acquiring complete set (cyclic scan) is show in supplementary figure 7.

Conventional scanning: The time required for data collection by conventional scanning scheme is given by

$$t_{tot/conv} = t_d + N \times (t_{exp} + t_r)$$

where, N is total #frames acquired on a single plane.

The table below (supplementary figure 7) shown the time required in the process of acquiring, M=10 plane collection with N=5000 frames in each plane. Control flow chart of traditional scan is depicted in supplementary figure 6.

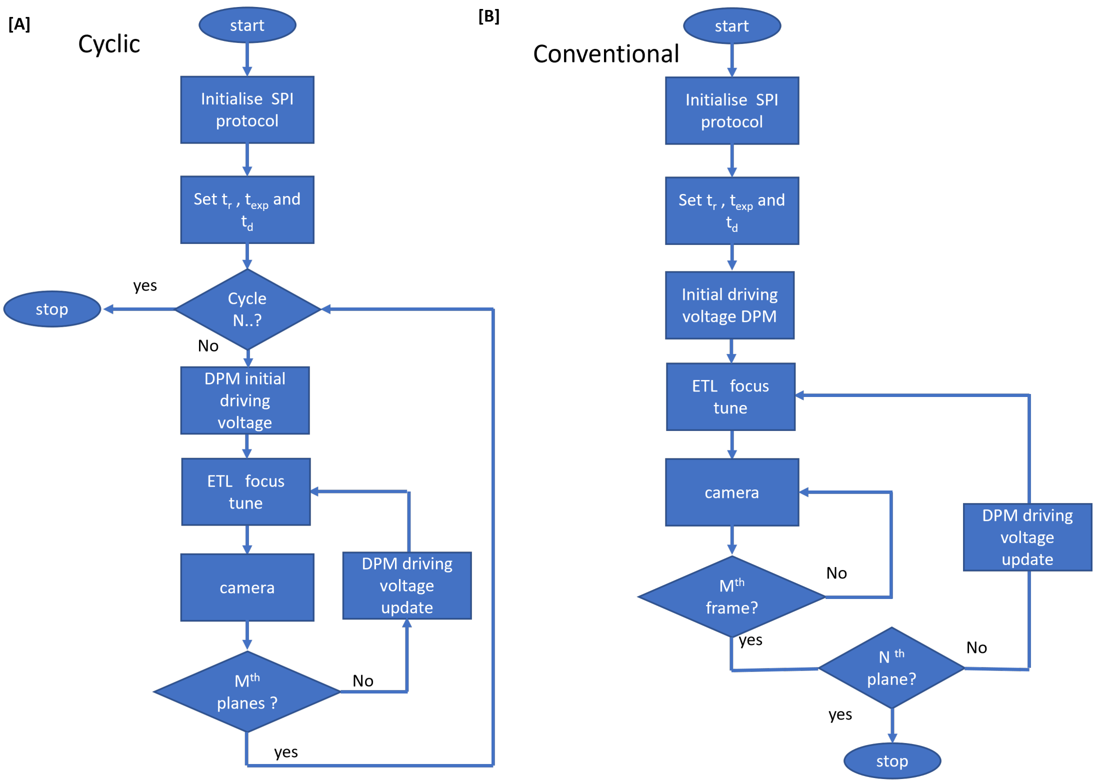

Supplementary Figure 6. Automation and acquisition control flow chart [A] cyclic scanning [B] conventional scanning.

T1

Parameters	Per cycle (ms)	Total time (min) $t_{tot}=(t_{exp}+t_r+t_d)\times M\times N$
Exposure(t_{exp})	300	54.16
Read time(t_r)	300	
Delay time(t_d)	50	

T2

parameters	Per plane(ms)	Total time (min) $t_{tot}=((t_{exp}+t_r)\times N+t_d)\times M$
Exposure(t_{exp})	150000	50
Read time(t_r)	150000	
Delay time(t_d)	50	

Supplementary Figure 7. Acquisition time for cyclic and conventional SMLM.

Supplementary Note 3. Volume Scanning Calibration of scanSMLM on Test Sample (fluorescent nano-beads)

Test sample preparation

Fluorescence nano sphere (170nm, 505/515nm, Invitrogen) is used to calibrate and standardised the system. 3µL of nanobead is thoroughly mixed with 3mL of 3% agarose gel at 45°C. Few drops of resultant mixture transferred to the live imaging disc (Mat Tek, Ashland, USA). The mixture is then allowed to cool and solidify for system calibration.

Z-shift calibration

The z-shift due to ETL tunning is calibrated using nano-beads disc placed on the object plane (specimen stage). A nano-bead (size ~175 nm) is focussed by the microscope and the corresponding mark is noted on the z-changing focus knob. Subsequently, the knob is rotated by the smallest division. This defocuses the image of nano-bead, which is again brought back by changing the ETL current / voltage (see, supplementary figure 8). So, by repeatedly changing the ETL current, a nano bead is focused-defocused-focused and z profile (see, main text, figure 3) is plotted. In this way, the calibration of ETL is accomplished. In principle, the ETL can achieve a smallest current of 0.07 mA which is about ~17.75 nm in the object plane for the *scanSMLM* detection configuration setup.

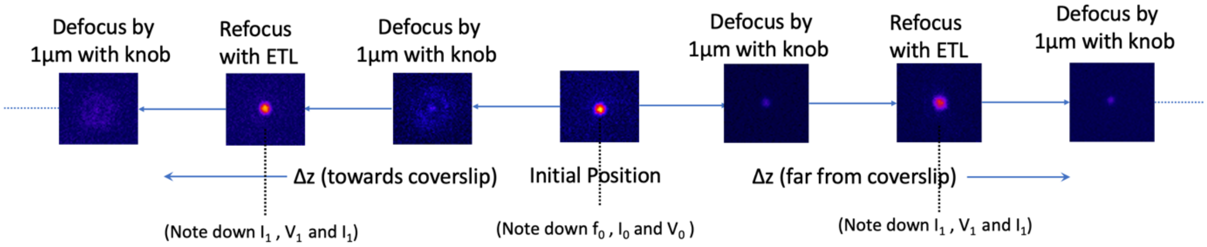

Supplementary figure 8. Z shift measurement is done repeatedly by measuring focusing and refocussing the PSF.

Cyclic volume scan and calibration

The repeatability of z scan position depends on two factors: repeatability of driving voltage and stability of ETL response. Repeatability of cyclic Z-scan position is calibrated using 170nm fluorescence nano beads (see, supplementary figure 9). Total 10 planes, each plane at a depth of 500nm florescence signal of nano beads are collected and scan cycle is repeated for N time (see, Supplementary Video **Supplementary Video 1**). The correlation coefficient of 0.948 with standard error 1.8 % among the 1st plane of each cycle indicated high degree of repeatability.

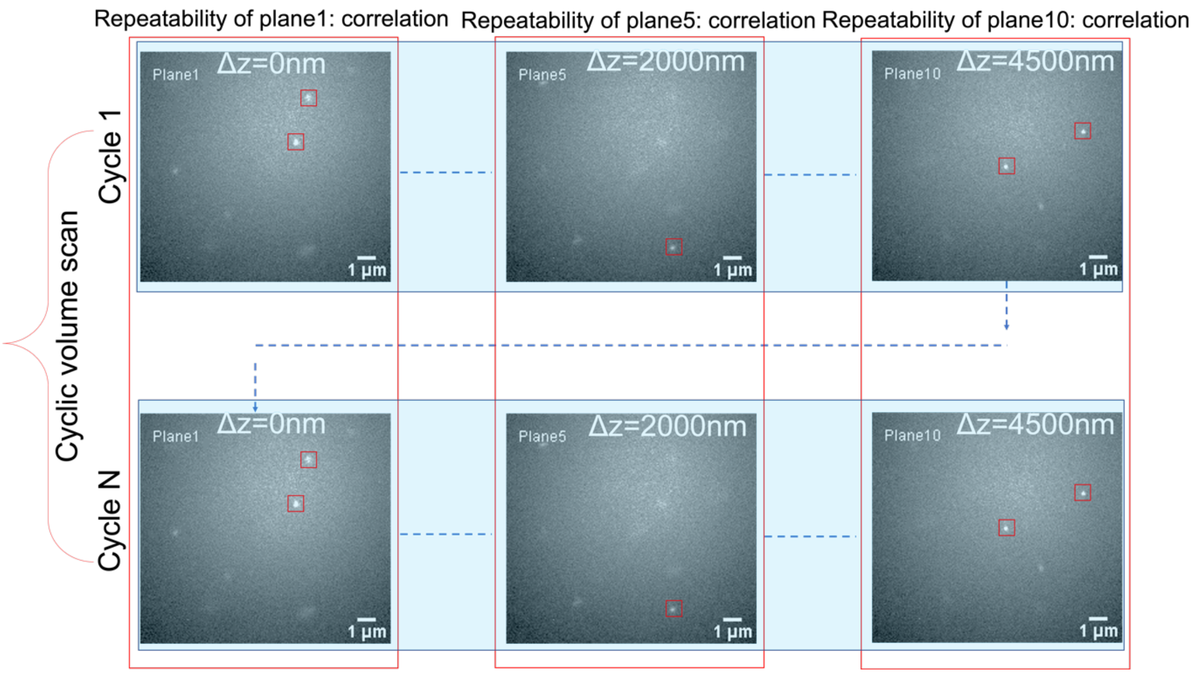

Supplementary figure 9. Repeatability of a particular plane (plane #1 in each cycle) over time during a cyclic scan.

The correlation coefficient (calculated using Pearson coefficient) used for the analysis is given by [2],

$$r = \frac{\sum_m \sum_n (A_{mn} - \bar{A})(B_{mn} - \bar{B})}{\sqrt{(\sum_m \sum_n (A_{mn} - \bar{A})^2)(\sum_m \sum_n (B_{mn} - \bar{B})^2)}}$$

where, $\bar{A}$ and $\bar{B}$ are image mean.

Supplementary Note 4. Cyclic Scanning of Actin Filaments in a Transfected NIH3T3 Cell

Actin filaments are volume scanned over the entire cell volume (10 z-planes). NIH3T4 cells were transfected with Dendra2-Actin plasmid DNA and incubated for 24 hours. Subsequently, the cells were fixed using standard protocol and imaged. All the reconstructed cell planes (10 planes) along with localization precision are shown in supplementary figure 10. Near constant localization precision throughout the cell volume suggests negligible effect on the detection efficiency at varying cell depths.

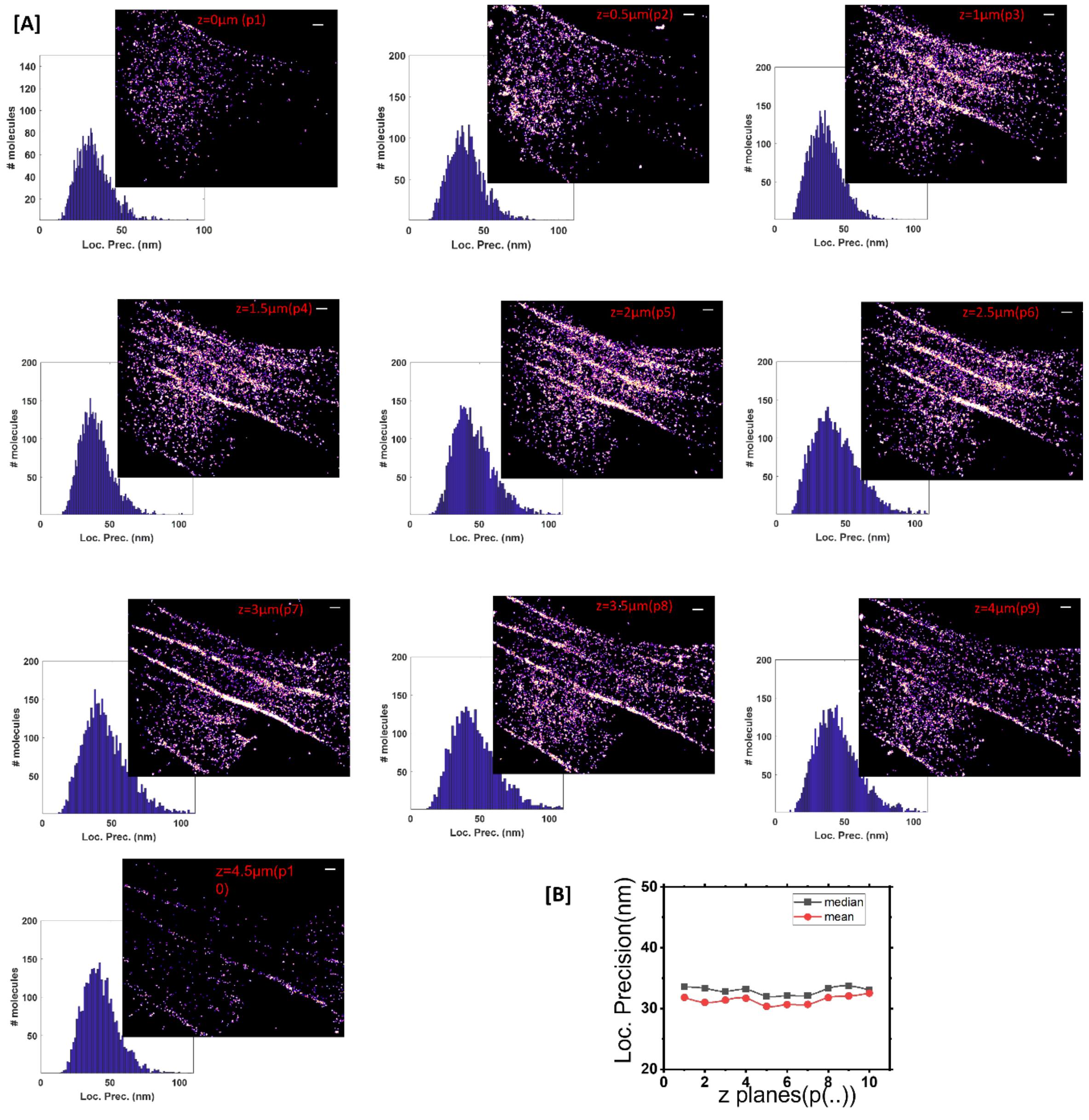

Supplementary Figure 10. [A] Cyclic scanning of Actin filaments up to a depth of 4.5micron. The super resolved images (10 planes) of Dendra2 tagged F-actin along with the localisation precession are shown. Spacing between two consecutive plane is 500nm. [B] Mean/ median of localisation at varying depths showing near-constant distribution throughout the cell volume.

Supplementary Note 5. Cyclic Scanning of Mitochondrial Network in a Transfected NIH3T3 Cell

Mitochondrial network and the distribution of mEos-Tom20 in a cell volume (10 axial planes). The NIH3T4 cells were transfected with nEos-Tom20 plasmid DNA and incubated for 24 hours. Subsequently, the cells were fixed, imaged and super-resolved images (for 10 planes) are reconstructed as shown in supplementary figure 11. This suggests near-constant distribution of single molecules at varying depths in the cell volume indicating negligible effect at large penetration depths.

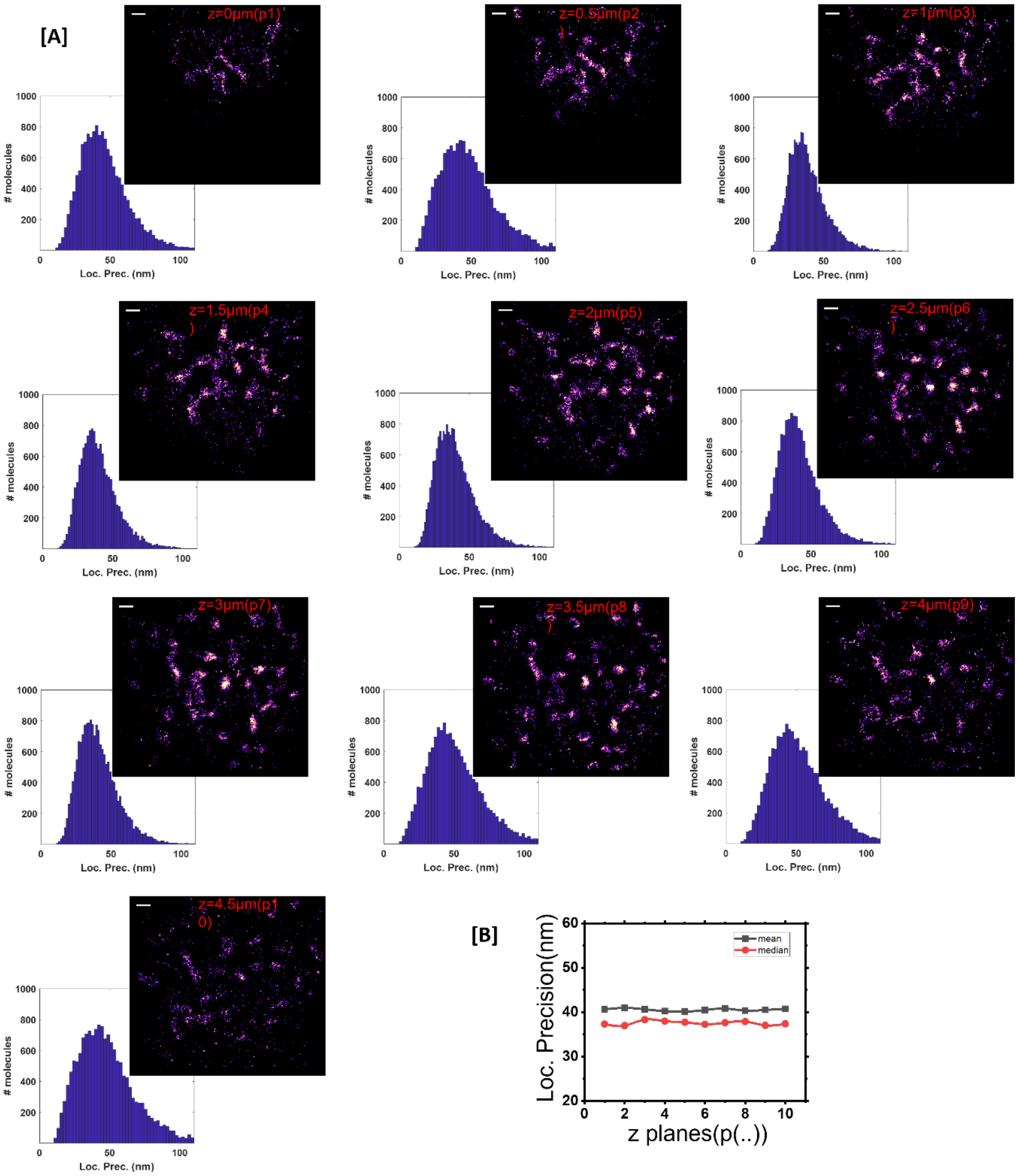

Supplementary Figure 11 Cyclic scanning of mitochondrial network up to a depth of 4.5micron. The super resolved image of MEOS 3.2 tagged TOM20 are shown along with the localisation precision. Spacing between two consecutive plane is 500nm. [B] Mean/ median of localisation at varying depths.

Supplementary Note 6. Cyclic Volume Scanning of Dendra2-HA in a Transfected NIH3T3 Cell

In the influenza-A model study, the distribution of Dendra2-HA molecules is studied across the cell volume (10 axial planes separated by 500 nm). Following standard protocol, NIH3T4 cells were transfected with Dendra2-HA plasmid DNA and incubated for 24 hours. Subsequently, the cells were fixed, imaged and super-resolved images were reconstructed. Reconstructions for data collected using cyclic and conventional scanning schemes are shown in supplementary figure 12 and 13, respectively.

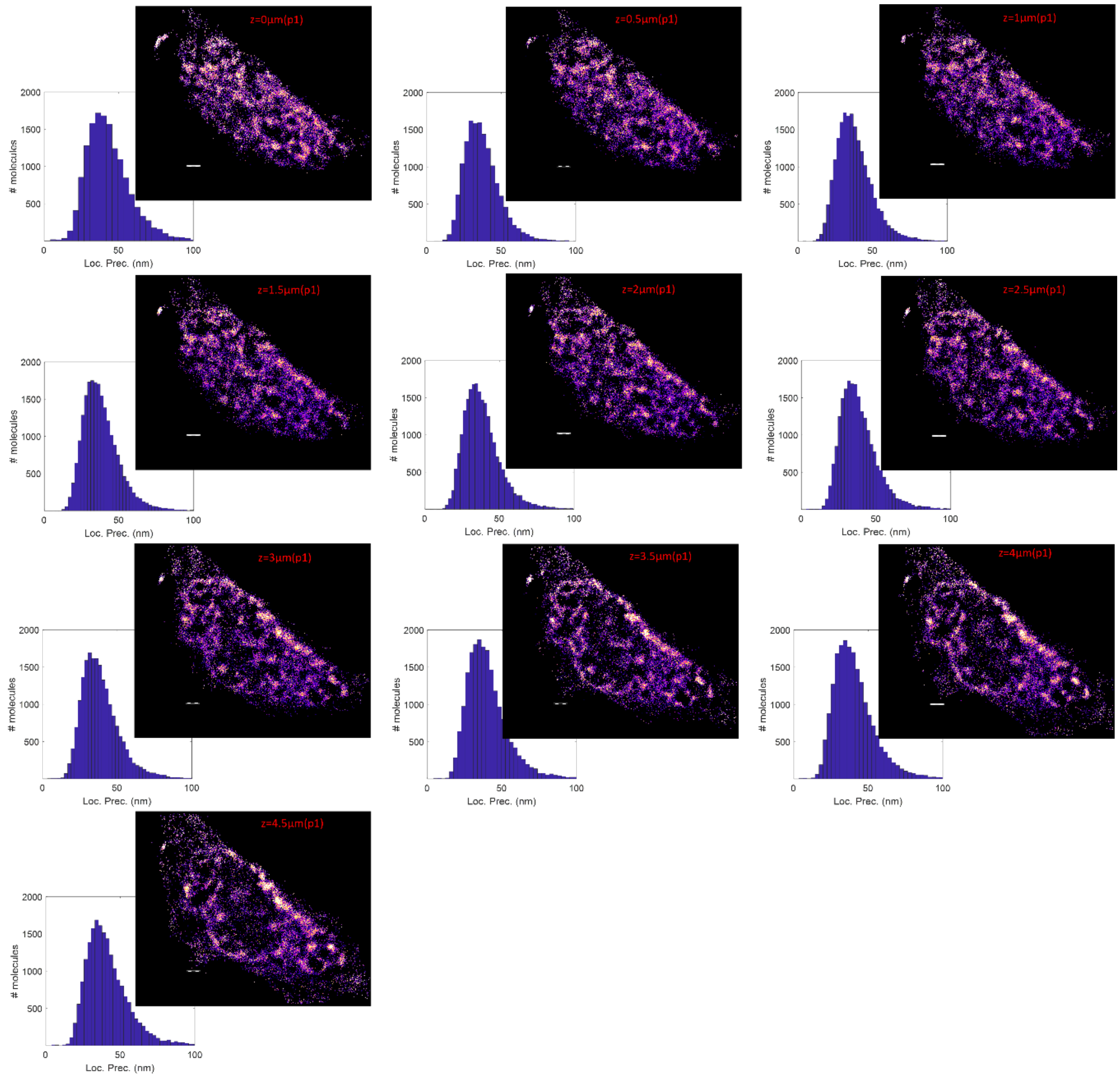

Supplementary Figure 12. Cyclic Scanning: Super resolved image of Dendra2-HA in cell volume and localisation precession. Scale bar = 1 µm.

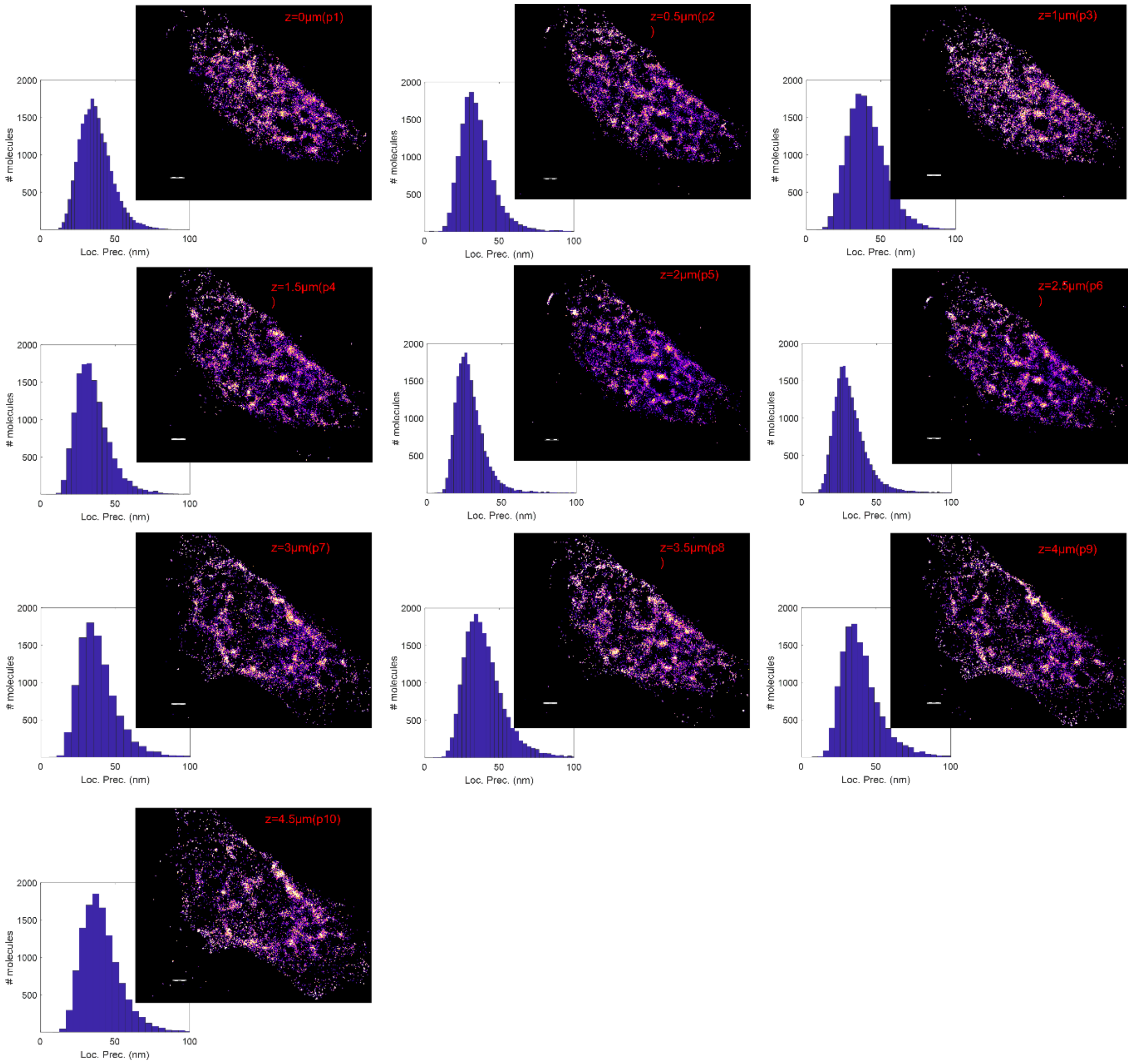

Supplementary Figure 13. Conventional Scanning: Super resolved image of Dendra2-HA in cell volume and localisation precession distribution. Scale bar = 1 µm.

In addition, cyclic scanning on another cell sample is carried out and the corresponding planes along with localization precision is shown in supplementary figure 14. The corresponding cell volume along with fluorescence and transmission images are shown in supplementary figure 15.

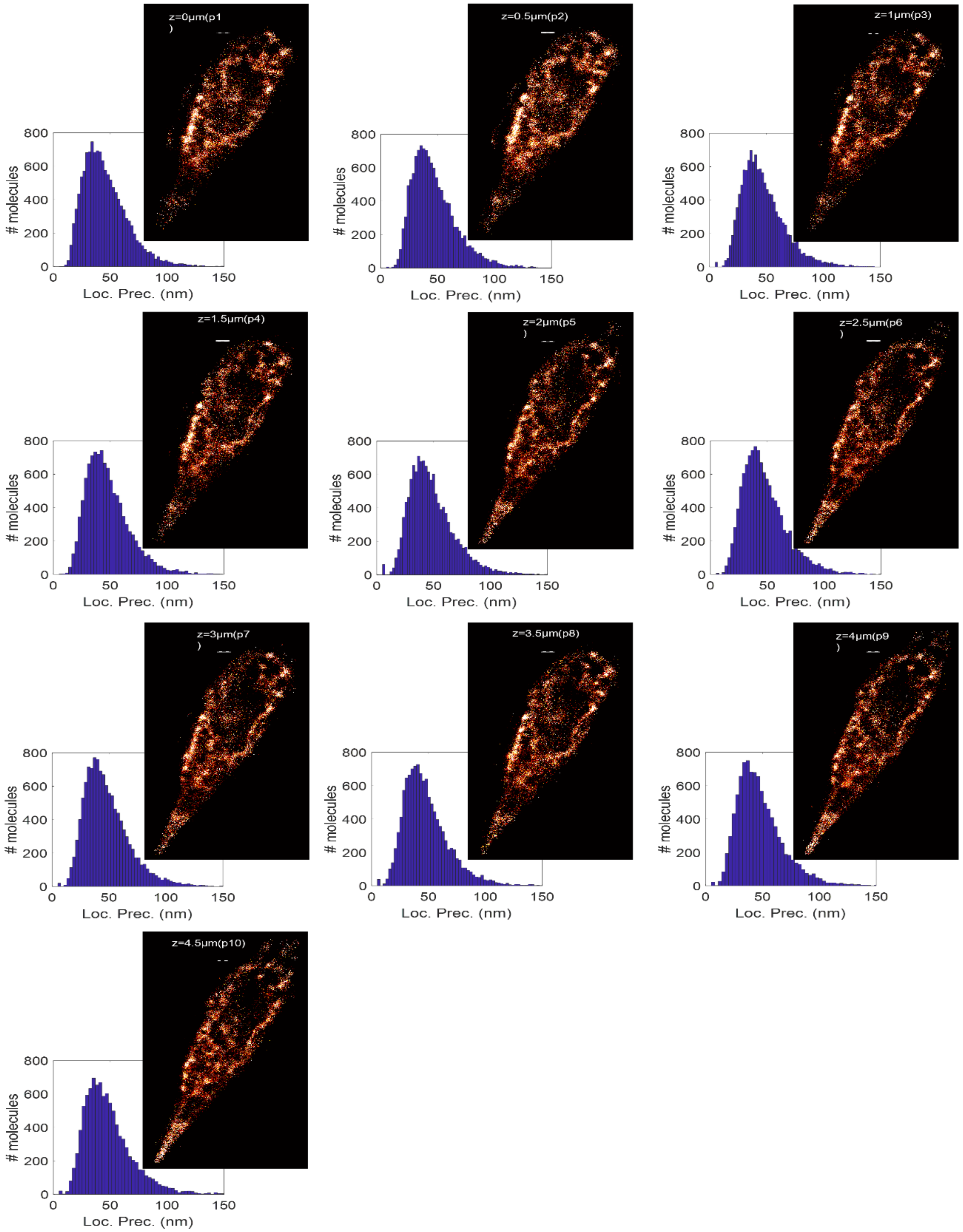

Supplementary Figure 14. Cyclic scanning of another cell (cell 2), along with localization precision.

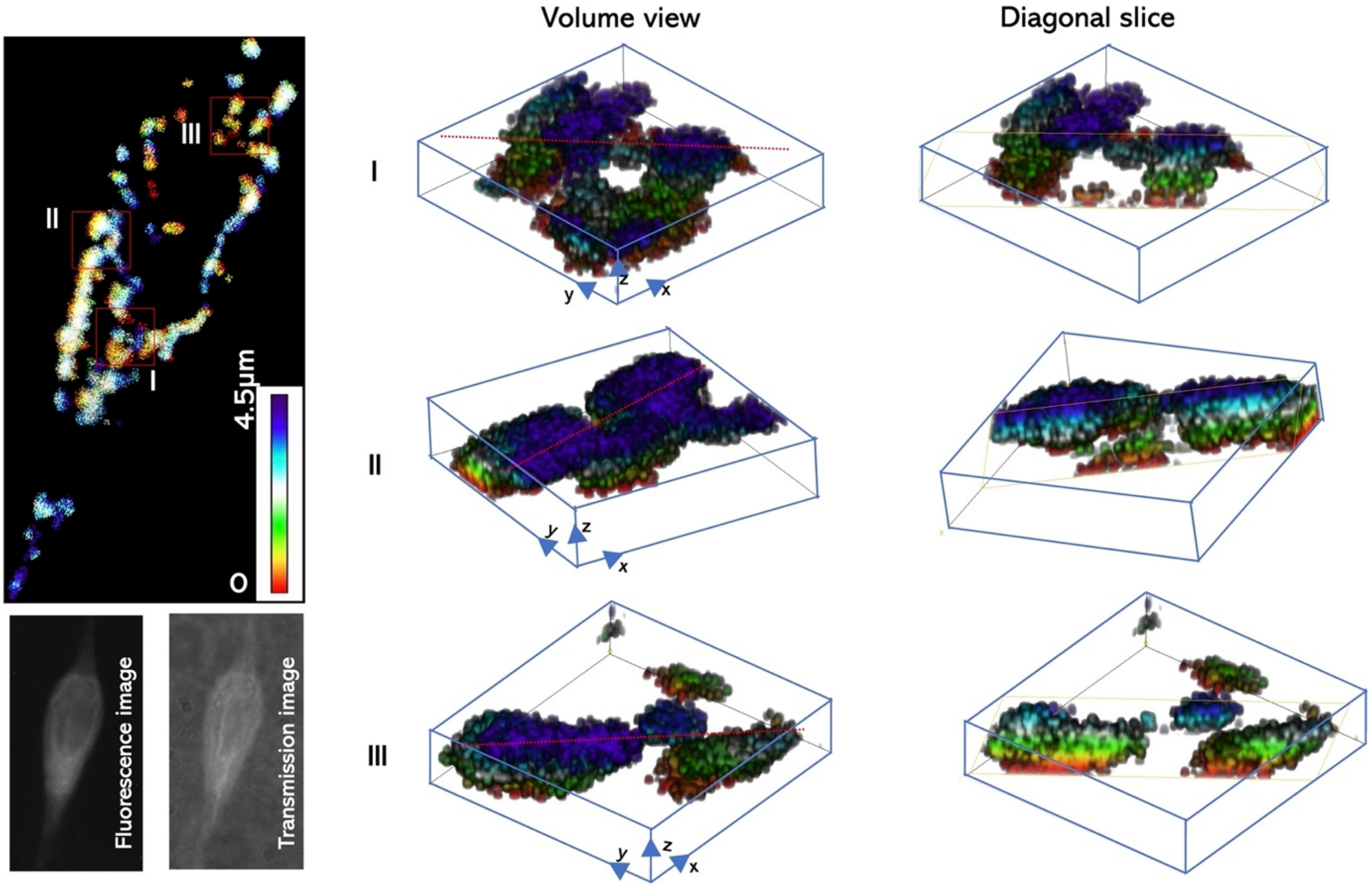

Supplementary Figure 15. Cell volumes (Volume and Diagonal views) and selected regions (I, II, III) of cell 2. Alongside, fluorescence and transmission images are also shown.

Supplementary Note 7. Point-based Clustering of Dendra2-HA Molecules in a Cell Volume

In Influenza-A, HA clustering play critical role, and provides vital information of heterogeneous and dynamic multi-molecular complexes [3]. Available clustering techniques have both advantages and disadvantages. Here, we employ another well-known clustering technique (hierarchical and point based clustering) for determining and analysing cluster formation. Supplementary figure 16 (cell 1) and supplementary figure 17 (cell 2) show point based clustering analysis that suggests the formation of discrete HA clusters throughout the transfected NIH3T3 cell volume as shown in clustered super-resolved map along with biophysical parameters (area, density and number of molecules per cluster) for the whole cell volume.

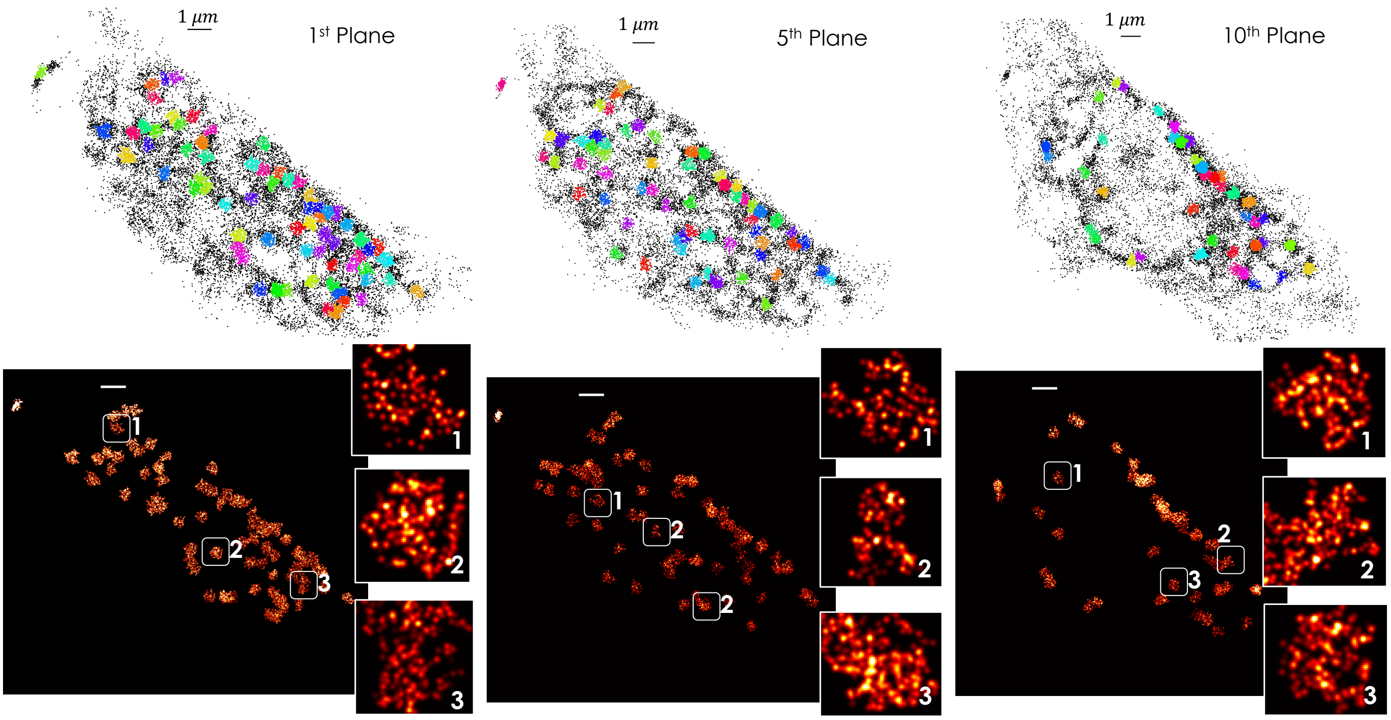

Biophys. Parameters	p1	p5	p10
Avg. area/cluster (μm^2)	.19	0.196	0.20
Avg. density/cluster (N/μm^2)	172	163.42	194
#avg mols./cluster (N)	893	823.52	923.63
Cluster fraction	0.202	0.171	0.103

Supplementary Figure 16. HA clusters identified by point-based clustering of cell 1 along with biophysical parameters. Corresponding biophysical parameters for cell 1 is also tabulated in adjoining table. Scale bar = 1 μm.

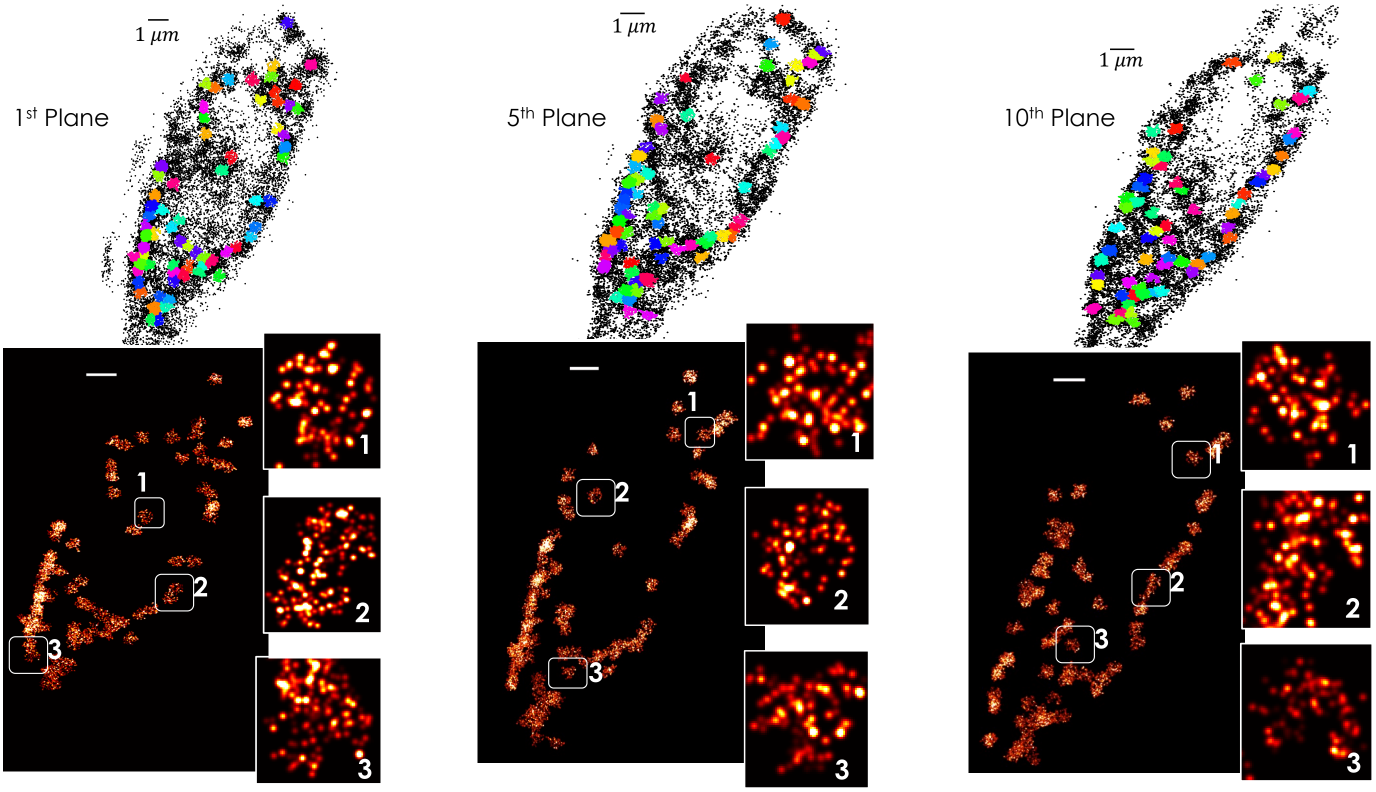

Biophys. Parameters	p1	p5	p10
Avg. area/cluster (μm^2)	.20	.2	.2
Avg. density/cluster (N/μm^2)	191	213.8	193.03
#avg mols./cluster (N)	936.9	1031.1	956.03
Cluster fraction	0.176	0.149	0.183

Supplementary Figure 17. HA clusters identified by point-based clustering in cell 2 along with biophysical parameters. Corresponding biophysical parameters for cell 2 is also tabulated in adjoining table. Scale bar = 1 µm.

Volume imaging and analysis give more information related to the critical feature of single molecule HA clusters in the cell volume. It is observed that the clusters are not limited to a single region / plane but span over several planes in a cell volume. Supplementary figure 18 gives the histogram of cluster span over more than 2 planes. It is observed that, approximately 5 clusters span over 5 planes (~2.5 µm) which is nearly half of the cell volume. Only a few clusters (~ 2) span across the entire cell volume.

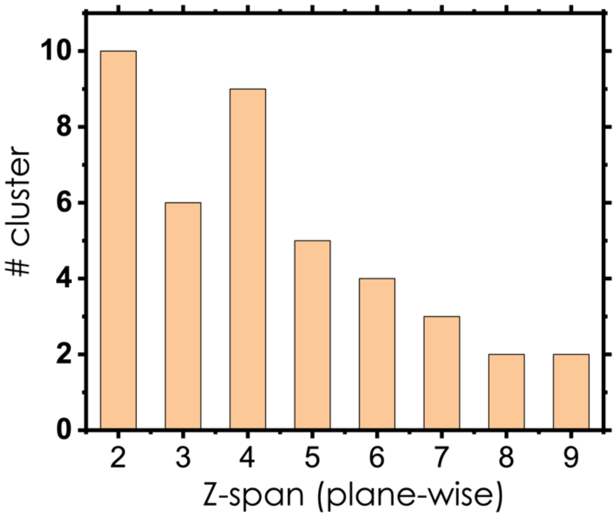

Supplementary Figure 18. Histogram of the span of clusters axially (along z-axis) in the cell volume. The spacing between two consecutive planes is 0.5 μm.

Supplementary Note 8. Confocal Study of HA Clusters

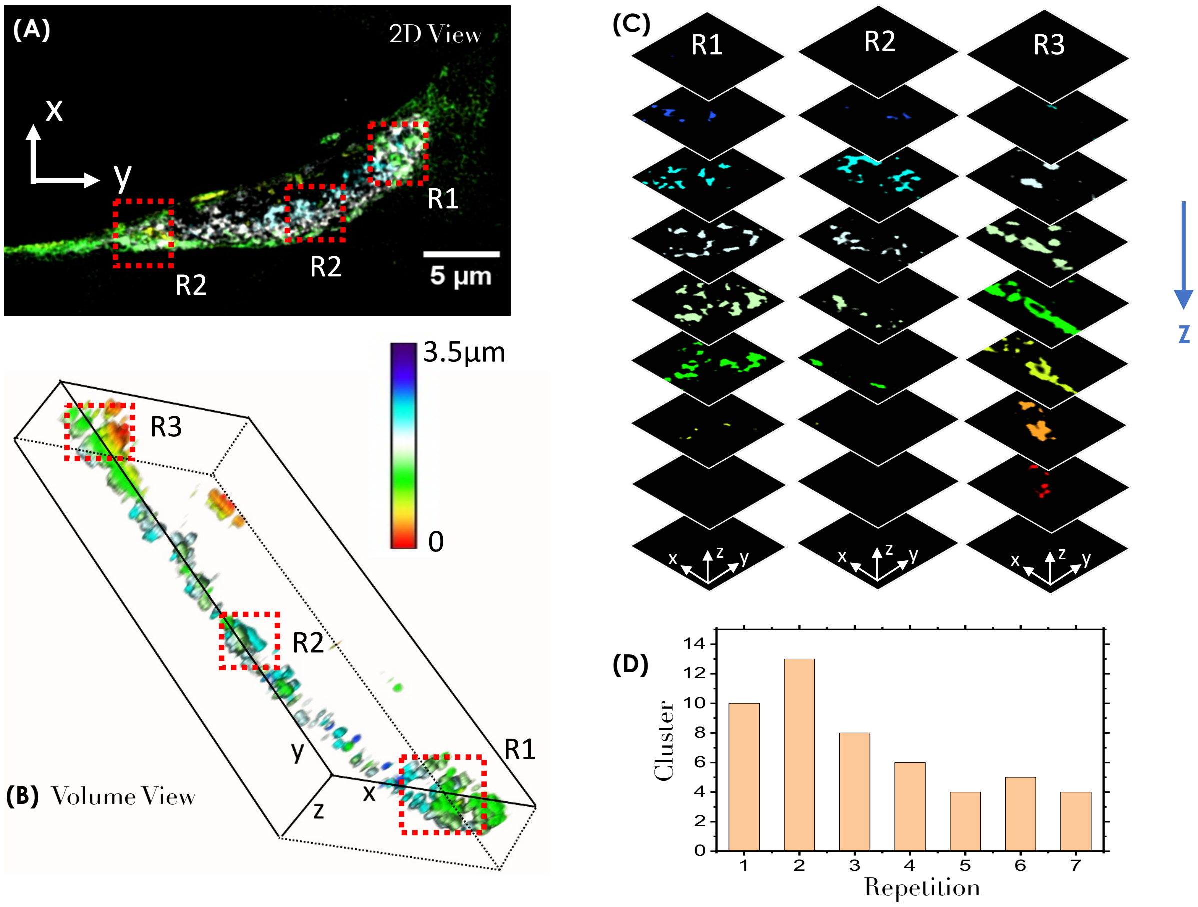

Supplementary Figure 19. Confocal Imaging: (**A**) Lateral view of Dendra2-HA transfected cell post 24 hrs transfection. (**B**) Rendered volume reconstructed from confocal image showing only HA clusters in a NIH3T3 cell. (**C**) Sectional images of three selected HA clusters (R1, R2, R3). (**D**) The histogram showing the number of clusters repeated in different planes, indicating connected HA clusters in cell volume.

In Influenza-A, the viral HA particles are known to form clusters in cellular system [4, 5, 6]. To understand the extent of cluster formation in the axial direction, scanSMLM is employed. Healthy NIH3T3 cells were transfected with Dendra2-HA plasmid DNA following the developed protocol as mentioned in the Methods section of main manuscript.

Supplementary figure 19 shows the confocal image (obtained using Leica SP8 Falcon (63X, 1.4 NA objective lens), Bioimaging Facility, IISc, Bangalore) of a transfected cell. Both lateral view and rendered volume of the cell are shown (A,B), which indicates the spread of HA clusters in the axial direction. This is better visualized in supplementary figure 19(C) where the clusters connected across the volume can be seen. A histogram of number of clusters vs repetitions indicate that a large number of clusters are span over more than single plane with a gradually decreasing repetitions. This clearly supports the studies performed using scanSMLM (main text, figure 6).

Supplementary Note 9. Confocal Study of Actin Filaments

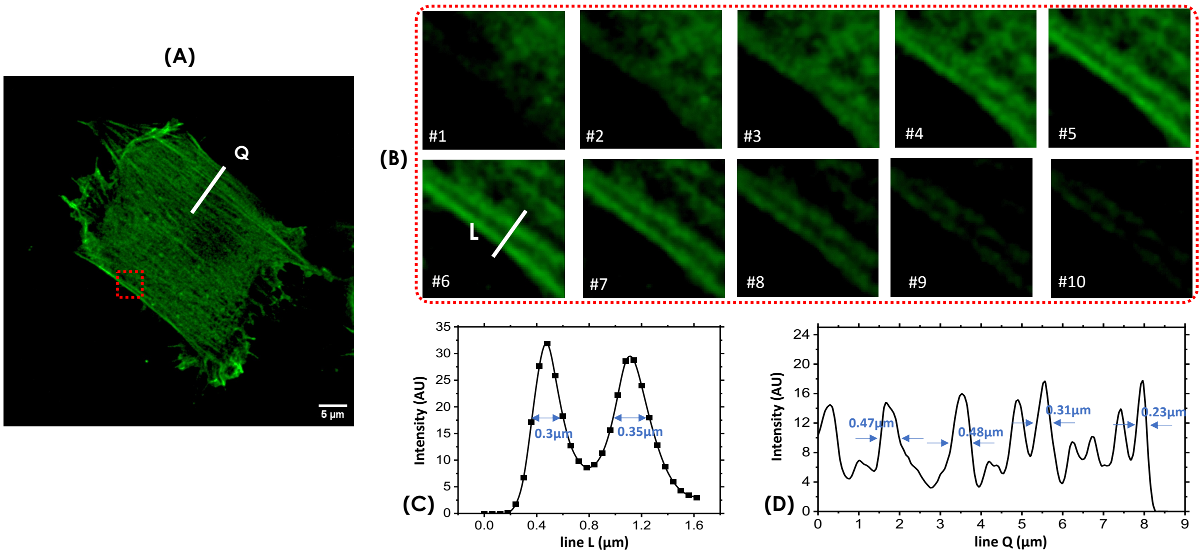

Supplementary Figure 20. (A) Confocal Imaging of Dendra2-Actin transfected cell post 24 hrs of transfection. (B) Enlarged view of a red dotted section along with all the z planes. (C, D) Line intensity plot along lines Q and L showing the size of actin bundles.

Confocal studies were performed to determine the size of Actin filaments / bundles along lateral and axial directions. Supplementary figure 20 shows the confocal image (obtained using Leica SP8 Falcon, Bioimaging Facility, IISc, Bangalore) of a transfected NIH3T3 cell post 24 hrs of transfection. The images were obtained with a pinhole size of 1 AU, and at a z-sampling of 270 nm. A high NA objective with a magnification of 63X and 1.4 NA was used. A light of wavelength 488 nm is used for excitation, and the fluorescence is collected at an emission wavelength (maximum) of 507 nm.

Supplementary figure 20A shows the structural composition of bundled Actin filaments in a NIH3T3 cell transfected. A small section of the image (marked by a red dotted rectangle) is enlarged, and all the z-sections are shown in supplementary figure 20B. Visually, a rough estimate of the extent of Actin bundles in the axial direction can be estimated, with the bundles approximately extending over 5 planes which is equivalent to ~ 1.35 mm. In addition, intensity plots are carried out along lines Q and L indicating the size of Actin-bundles to be ~ 0.44 mm. However, we noted that the Actin bundles vary over a 0.23 – 0.48 mm range. This is consistent with the dimension of Actin bundles determined using *scanSMLM* system.

Supplementary Note 10. FRC Analysis

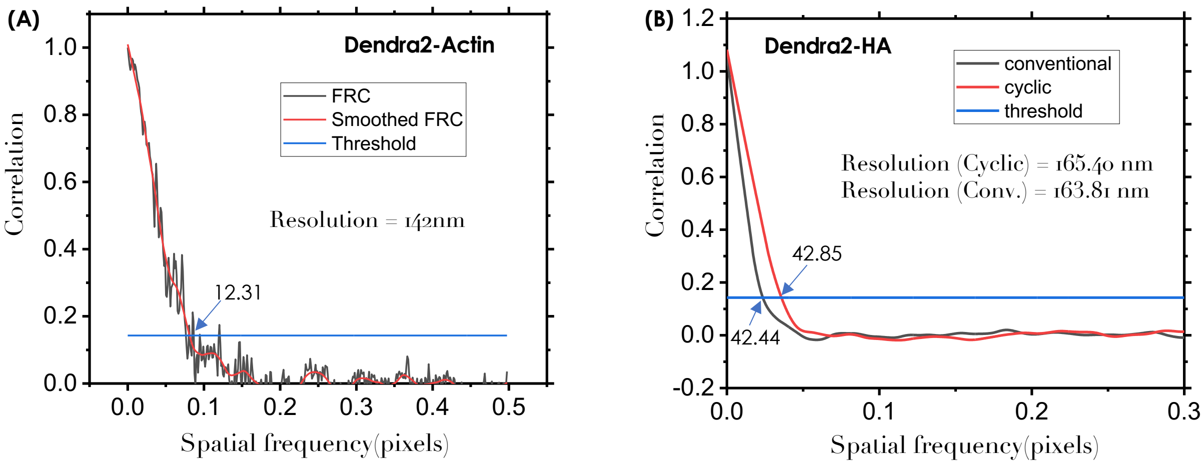

Supplementary Figure 21. FRC Analysis: (A) FRC analysis of Actin filaments in Dendra2-Actin transfected NIH3T3 cells, (B) FRC analysis of HA clusters in Dendra2-HA transfected cells for both Cyclic and Conventional scanning methods are shown.

Fourier Ring Correlation (FRC) is extensively used to quantify the image resolution for optical microscopy. Specifically, this technique is preferred for localization microscopy since this heavily depends on the localization uncertainty and density of single fluorescent labels [7, 8, 9]. The analysis involves dividing a super-resolution image (set of single emitter localizations) into two independent subimages (statistically independent subsets of emitters), followed by determining the statistical correlation of their Fourier transforms (in the frequency domain). Accordingly, FRC is calculated using the following expression,

$$FRC = \frac{\sum_{r\epsilon r_i} F_1(r).F_2(r)^*}{\sqrt{\sum_{r\epsilon r_i} F_1^2(r).\ \sum_{r\epsilon r_i} F_2^2(r).}}$$

where, F_1 and F_2 are Fourier transform of two subimages and r_i is i^{th} frequency bin.

The FRC curve approaches unity for low spatial frequencies, whereas it goes to zero for high frequencies, which is predominantly due to noise. The inverse of the spatial frequency at which the FRC curve drops below the threshold determines the image resolution. For the present analysis, a fixed threshold of 0.143 is used [8, 10]. To generate the FRC curve, we used Fiji plugin and selected 1/7=0.143 as a threshold. The corresponding FIRE number (a correlation value when the FRC curve reaches 0.143) is determined, and image resolution is calculated. Supplementary figure 21 shows the FRC analysis for Actin filaments and HA clusters in Dendra2-Actin and Dendra2-HA transfected cells, respectively. This indicates a resolution of 142 nm for Actin filaments, whereas HA clusters were resolved at 165.40 nm and 163.81 nm for cyclic and conventional schemes, respectively.

Supplementary Note 11. Noise Analysis

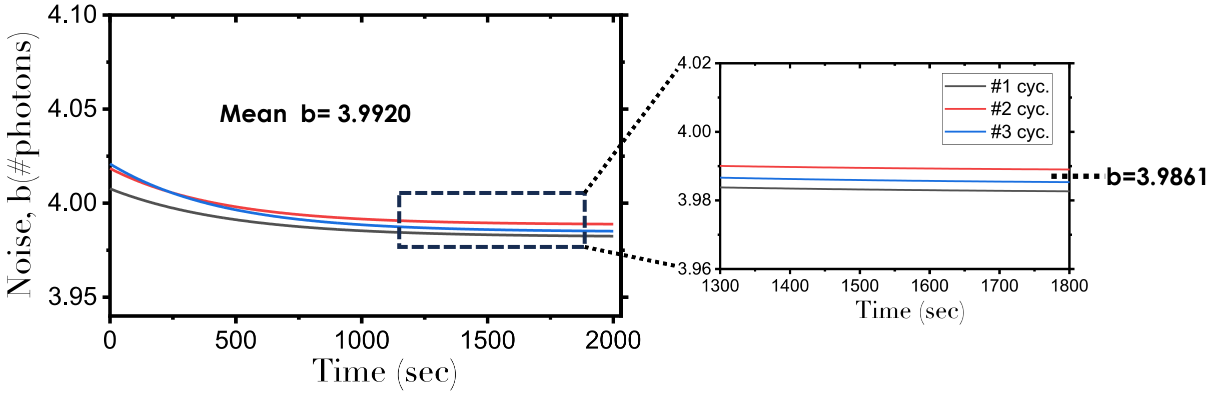

Supplementary Figure 22. Noise versus time for the cyclic scheme. The noise is calculated for three chosen planes (#1, #2, #3). The inset shows near-constant noise at large time.

Noise is an important parameter for calculating localization precision and needs careful study over time, specifically for long-time imaging [11]. The noise is calculated over the data acquisition time, as shown in supplementary figure 22. To substantiate, we choose three different planes (#1, #2, #3) acquired using cyclic scheme. It is evident that noise shows exponential decay over time, with an overall average of 3.99. Alongside, an enlarged section of the plot is shown for large acquisition time, which indicates saturation of noise with an average of 3.9861 and a variance of < 0.001. Low average noise, along with a negligible variance, suggests that scanSMLM is suitable for long-time imaging.

Supplementary References:

[1] Adaptable single molecule localization microscopy (aSMLM) for super resolution optical fluorescence imaging Appl. Phys. Lett. 119, 173703 (2021).

[2] J. P. Lewis, Fast Template Matching, Vision Interface, 120-123, 1995.

[3] E. Sherman, Resolving protein interactions and organization downstream the T cell antigen receptor using single-molecule localization microscopy: a review. Methods Appl. Fluoresc. 4, 22002 (2016).

[4] N. M. Curthoys et al., Influenza Hemagglutinin Modulates Phosphatidylinositol 4,5-Bisphosphate Membrane Clustering, Biophys. J. 116, 893–909 (2019).

[5] P. Chlanda, and J. Zimmerberg, Protein–lipid interactions critical to replication of the influenza A virus, FEBS Lett. 590, 1940 (2016).

[6] S. T. Hess, Gould, T.J., Gudheti, M.V. et al., Modern fluorescent proteins: from chromophore formation to novel intracellular applications, Proc. Natl. Acad. Sci. USA 2007, 104, 17370–17375.

[7] G. Tortarolo, Castello, M., Diaspro, A., Koho, S. & Vicidomini, G. , Evaluating image resolution in stimulated emission depletion microscopy, Optica 5, 32–35 (2018).

[8] R. P. J. Nieuwenhuizen et al. , Measuring image resolution in optical nanoscopy, Nat. Methods 10, 557–562 (2013).

[9] N. Banterle, Bui, K. H., Lemke, E. A. & Beck, M. , Fourier ring correlation as a resolution criterion for super-resolution microscopy, J. Struct. Biol. 183, 363–367 (2013).

[10] R. Beckmann et al. Alignment of conduits for the nascent polypeptide chain in the ribosome-Sec61 complex, Science 278, 2123–2126 (1997).

[11] Thompson, R. E., Larson, D. R. & Webb, W. W. Precise nanometer localization analysis for individual fluorescent probes. Biophys. J . 82, 2775–2783 (2002).
