# Supplementary material for: Scanning single molecule localization microscopy (scanSMLM) for super-resolution volume imaging
Source: Commun Biol. 2023 Oct 17;6:1050. doi: 10.1038/s42003-023-05364-2 (PMC10582190; doi:10.1038/s42003-023-05364-2)
Supplement: Supplementary file 2 — Description of Additional Supplementary Files [file 42003_2023_5364_MOESM2_ESM.pdf]

Description of Additional Supplementary Files

File name: Supplementary Data 1
Description: Numerical source data underlying the graphs, tables and charts presented in the main figures.

File name: Supplementary Video 1
Description: Cyclic scanning of nano beads for calibration.

File name: Supplementary Video 2
Description: Cyclic scanning of micro beads (test sample).

File name: Supplementary Video 3
Description: Reconstructed 3D map of Dendra2-Actin molecules on Actin filaments.

File name: Supplementary Video 4
Description: Reconstructed 3D map of mEos-Tom20 molecules on the mitochondrial network.

File name: Supplementary Video 5
Description: Recorded super-resolution data (cyclic scan) of Dendra2-HA transfected cell.

File name: Supplementary Video 6
Description: Conventional scanning Dendra2-HA transfected cell.

File name: Supplementary Video 7
Description: 3D cluster of Dendra2-HA molecules.
